# Characterisation of a cohort of opportunistically recruited patients with COVID-19 and approaches to patient stratification

**DOI:** 10.1186/s12879-026-13070-7

**Published:** 2026-03-31

**Authors:** Shaufa Shareef, Eleanor Matthews, Joseph Dodds, Alasdair Silverberg, Matthew E. Daly, Waqar Ahmed, Jonathan Bannard-Smith, Lee A. Gethings, Adam King, Chris Hughes, Stephen Fowler, Timothy Felton, Angela Simpson, E. N. C. Mills

**Affiliations:** 1 Division of Immunology, Immunity to Infection and Respiratory Medicine, School of Biological Sciences, Manchester Institute of Biotechnology, Manchester Academic Health Sciences Centre, University of Manchester, Manchester, Greater Manchester, M1 7DN UK; 2 Manchester Royal Infirmary, Manchester University NHS Foundation Trust, Manchester, Greater Manchester, M13 9WL UK; 3 Present Address: Waters Corporation, Stamford Avenue, Altrincham Road, Wilmslow, Cheshire, SK9 4AX UK; 4 School of Biosciences and Medicine, the University of Surrey, Guildford, Surrey, GU2 7XH UK; 5 Wythenshawe Hospital, Manchester University NHS Foundation Trust, Manchester, Greater Manchester, M23 9LT UK

**Keywords:** COVID-19, Clinical cohort, Severity, Biomarkers, Proteomics, Lipidomics

## Abstract

Multiomic biomarker discovery generally uses well curated cohorts of patients and health individuals, although this can present challenges in validation and deployment of biomarkers for diagnosis in “real world” situations. An alternative approach is to use cohorts of patients in routine clinical care, but this necessitates identification of relevant comparator groups. In infectious disease one option is to compare patients with a current infection with those who are convalescent. This approach has been applied using opportunistically collected biological samples from the Manchester Allergy, Respiratory and Thoracic Surgery Biobank COVID-19 cohort unimmunised hospitalised or convalescent patients for whom extensive clinical data and classical biomarker analysis are available. Pilot serum lipidomic and proteomic profiling was undertaken employing novel, rapid mass spectrometry methods. The cohort comprised 222 individuals, 25% of whom were of non-white ethnicity, 68% of whom were male and 66% were either overweight or obese. Around half (*n* = 116) were classified as severe based on symptomology using the WHO score, with a patient management score (the Manchester Severity Score) splitting the WHO “critical” category into two further sub-categories. Stratification of patients into those who had a current infection or were convalescent using both severity scores provided consistent results in analysis of classical cellular and biochemical markers. For example, C-reactive protein (CRP) was higher (mean 74.5 mg/L) in those with a current infection compared to those who were convalescent (mean 35.1 mg/L). Pilot multiomics analysis using novel methodology showed good reproducibility and identified lipid and protein biomarkers previously observed in COVID-19 infections including phosphatidyl cholines, triglycerides and C-reactive protein. The relative quantification of CRP by proteomics was correlated with conventional measurements. These data demonstrate the feasibility of using samples from patients who have either a current infection, or who are convalescent, as comparators and that the multiomics analysis pipeline is suitable for wider lipidomic and proteomic analysis in future.

**Supplementary Information:**

The online version contains supplementary material available at 10.1186/s12879-026-13070-7.

## Highlights

Describes an ethnically diverse “real world” cohort of unimmunised COVID-19 patients with opportunistically collected serum and plasma samples.

Demonstrates the feasibility of undertaking multiomics biomarker discovery by stratifying patients by infection status.

Effective application of sample handling protocols and novel rapid mass spectrometry methodology with good reproducibility.

**Supplementary Information:**

The online version contains supplementary material available at 10.1186/s12879-026-13070-7.

## Background

Biomarker discovery studies using multiomics approaches can provide insights into the molecular basis of disease and identify candidate diagnostic markers. Such studies have historically sought to use carefully selected patient populations, but there can be difficulties in then applying candidate markers in real world situations. Whilst opportunistically collected samples are highly heterogeneous, they are a better reflection of patient populations encountered in routine clinical practice. The impetus provided by the COVID-19 pandemic and the need to understand the response of a naïve population to a novel viral pathogen resulted in the collection of many biological samples from infected individuals. However, the immense burden placed on healthcare systems by the pandemic mitigated against the collection of samples in a highly structured manner, as might ordinarily be the case. This has hampered the application of multiomics methods to identify disease biomarkers and better understand the immune responses underlying severe COVID-19 [1–3]. For example, a lack of clinical metadata in many studies means patient classification has been simplistic, often consisting of, for example, “hospitalised” and “non-hospitalised” patients and an inability to differentiate patients with mild COVID-19 disease who were hospitalised due to unrelated conditions. Research in the early stages of the pandemic was also often limited by smaller population sizes and/or a focus on targeted immunoproteomics [1–5] with larger studies generally being undertaken in association with therapeutic and vaccine trials [6–10].

This report describes approaches to using opportunistically collected biological samples from hospitalised and convalescent patients obtained from the Manchester Allergy, Respiratory and Thoracic Surgery (ManARTS) Biobank. There were collected during the pandemic and for which a large amount of systematically collected patient metadata is available. It explores the feasibility of approaches to patient stratification where convalescent patients were used as a comparator to those with current, active, infections of different severity rather than the classical use of age- and sex-matched healthy volunteers as comparators. This was achieved through an analysis of the general characteristics of the population and conventional disease biomarkers. In addition, the sample preparation workflows applied in lipidomics and proteomics analysis, using novel, rapid, discovery liquid chromatography mass spectrometry (LC-MS-MS) methodology employing rapid (11 min) analysis time, were assessed for their utility and reproducibility.

## Methods

### Cohort description

The cohort consists of 222 participants recruited between March 2020 and January 2021 to the ManARTS Biobank, which is funded by the National Institute for Health Research (NREC 15/NW/0409). Participants came from two hospitals that form part of the Manchester University NHS Foundation Trust, Wythenshawe hospital and Manchester Royal Infirmary. Biological samples were opportunistically collected and included samples taken close to hospital admission, during a patient’s hospital stay, and/or at follow up appointments. Sample processing to provide serum or plasma was performed as far as practicable, using standard procedures. On occasion, where it was not possible to take a bespoke blood sample for the Biobank, the plasma and/or serum remaining after routine testing was stored and for which there may have been delays in freezing. All samples of plasma or serum were stored at -70 °C until being prepared for analysis.

### Data curation

Anonymised demographic and clinical data was drawn from patient records and curated for further analysis using R 4.3.1 [11] and the *tidyverse* [12] and *XLConnect* packages [13]. Statistical analysis were carried out using R 4.1.0 [11], the *rstanarm* [14] and the *report v0.2.0* [15] packages.

In some instances, numeric data fields also contained text characters; these were removed during the preparation of the data analysis-ready format. Biologically implausible test results were investigated and those values that were clearly a typographical error or the result of a non-standard unit being used were corrected. Where test results were incompatible with life (e.g. respiratory rate = 1), but no correct value was apparent, these values were removed. Where the same data were sourced from clinical records in two different ways they were combined to give a more complete dataset; where there were mismatches, the more complete source dataset was used. Clinical data such as chest X-ray grading, comorbidities, symptoms on admission, drug treatments, sepsis and septic shock, intensive care unit (ICU) admission, and thrombotic events present in free text fields were extracted and encoded into a data-analysis ready format.

The severity of COVID-19 in the cohort was scored either using the in-house Manchester COVID-19 Severity Score (MCSS [16–19]; Figure S4) or retrospectively using the World Health Organisation (WHO) severity score [20] (Figure S3). In some instances, the MCSS had either not been documented or were documented inconsistently in the data extract. The MCSS for these patients was recalculated based on data in the extract regarding use of invasive ventilation or based on the amount of oxygen they were given.

As samples taken for multiomics analysis were not necessarily during a patient’s active infection, patients were further stratified into two groups, active infection and convalescent, based on their diagnosis date (Figure S5). Diagnosis date was defined as first positive swab, where available, or diagnosis by a CVCX1 grade chest X-ray [21] along with COVID-typical symptoms. Diagnosis date was used rather than symptoms onset as patient-reported symptoms onset date due to the non-specificity of symptoms documented and unreliability of patient-recall [22]. Active infection was defined as ≤ 14 days from diagnosis to sample; convalescence was defined as ≥21 days from diagnosis to sample and sample collection after discharge, based on the potential for isolation of replication-competent virus [23–25]. Patients with an unclear classification (between 15 and 20 days, or after 20 days but still hospitalised) were stratified into these groups by assessing their clinical picture in terms of documented severity score, immunosuppression, and significant clinical events, including ICU admission and death.

### Proteomics analysis

A pooled quality control (QC) sample was prepared from patient plasma samples. 7.5 µL of plasma was diluted with 10mM pH 7.4 phosphate buffered saline (PBS) for a final volume of 10 µL. The fourteen most abundant plasma proteins (i.e., albumin, IgG, IgA, IgM, IgD, IgE, kappa and lambda light chains, alpha-1-acidglycoprotein, alpha-1-antitrypsin, alpha-2-macroglobulin, apolipoprotein A1, fibrinogen, haptoglobin, and transferrin) were depleted using High Select™ Depletion Spin Columns (Thermofisher Scientific Ltd). Depleted samples were processed in duplicate aliquots by adding 7.5 µL RapiGest™ SF Surfactant (Waters Corporation), then reducing with 4.1 µL 100 mM dithiothreitol for 10 min at 80 °C, alkylating with 5.5 µL 250 mM iodoacetamide for 30 min in the dark, then diluting with 21.6 µL 250 mM ammonium bicarbonate. 100 µg/mL Trypsin Gold (Promega) was prepared by diluting in 50 mM ammonium bicarbonate. Samples were digested overnight at 37 °C, then quenched with 2.2 µL 25% formic acid (v/v), and desalted with solid-phase extraction using OASIS HLB 96-well plate (Waters Corporation). Wells were conditioned with 200 µL acetonitrile followed by 200 µL water, loaded with 100 µL of sample, then washed with 800 µL 0.1% trifluoroacetic acid (v/v) followed by 200 µL water. A vacuum pump was used to elute the waste liquid after each step. Bound peptides were eluted with 50 µL 70% acetonitrile (v/v), then evaporated under nitrogen and reconstituted with 20 µL of 0.2% acetonitrile (v/v), 0.05% formic acid (v/v). Low protein-binding plastics were used for all tubes and plates. All reagents used were high performance liquid chromatography (HPLC)-grade.

One-dimensional nanoscale liquid chromatography (LC) separation of tryptic peptides was performed with an ACQUITY Premier Ultra-performance liquid chromatography (UPLC) (Waters Corporation, Milford, MA, USA), equipped with an ACQUITY Premier CSH C18 1.7 μm, 2.1 mm x 100 mm analytical reversed-phase column (Waters Corporation, Milford, MA, USA). Mobile phase A consisted of water containing 0.1% (v/v) formic acid, whilst mobile phase B was acetonitrile containing 0.1% (v/v) formic acid. Peptides were separated using a gradient of 5–35% mobile phase B over 15 min at a flow rate of 150 µL/min, followed by 85% wash prior to re-equilibration, for a total runtime of 23 min. The analytical column temperature was maintained at 55 °C. Lock mass solution was delivered by the auxiliary pump of the LC system at 5 µL/min to the reference sprayer of the source of the mass spectrometer. Mass spectrometric analysis was performed using a SELECT SERIES Cyclic Ion Mobility mass spectrometer (Waters Corporation, Wilmslow, United Kingdom). For all measurements, the mass spectrometer was operated in v-mode with nominal resolution of 50,000 full width at half-maximum (FWHM). All analyses were performed in positive mode electrospray ionisation (ESI). The ion source block temperature and capillary voltage were set to 100 °C and 2.2 kV, respectively. The time-of-flight analyser of the mass spectrometer was externally calibrated with a NaCsI mixture from *m/z* 50 to 1990. The data were post-acquisition lock mass-corrected using the doubly charged monoisotopic ion of [Glu1]-Fibrinopeptide B, at m/z 785.8421. The reference sprayer was sampled with a frequency of 120 s. Accurate mass LC-MS data were collected in a randomised order using the high definition mass spectrometry (HDMS^E^) mode of acquisition [26, 27]. The spectral acquisition time in each mode was 0.15 s with a 0.02 s interscan delay. In low energy MS mode, data were collected at constant trap and transfer collision energy of 6 eV (per unit charge). In the elevated energy mode, the trap collision energy was ramped from 19 eV to 45 eV (per unit charge) in 0.15 s. One cycle of low and elevated energy data was acquired every 0.3 s.

Proteins were identified based on a 1% false discovery rate (FDR) using Progenesis QI for proteomics. Peaks were aligned using a reference selected from the pooled quality control (QC) sample, detected peptides filtered for charge between + 2 and + 6, and searched against UniProtKB reviewed human proteins (20,386 sequences). In silico parameters accounted for trypsin digestion with ≤ 2 missed cleavages, with modifications set as cysteine carbamidomethylation (fixed), and methionine oxidation, asparagine/glutamine deamidation, and serine/threonine/tyrosine phosphorylation (variable). Peptide and fragment tolerance was set as 10 and 20 ppm respectively, with a minimum of one fragment per peptide, three fragments per protein and one unique peptide per protein required for identification. Peptides with a score < 5 were removed. Relative quantitation was performed using Hi-3 [28], with data normalised to all proteins. Further quality control was performed in R 4.2.1 by selecting proteins present in at least two technical replicates and in both biological replicates.

### Lipidomics analysis

A pooled quality control (QC) sample was prepared from patient serum samples. Samples were processed in duplicate by adding 15 µL of 1 in 10 dilution of SPLASH^®^ LIPIDOMIX^®^ Mass Spec Standard (Avanti Polar Lipids) in chilled isopropanol along with 63 µL methanol to 30 µL serum, followed by 250 µL methyl tertiary-butyl ether (MTBE). Samples were vortexed for 15 s, then incubated for 1 h at 4 °C, after which 138 µL water was added and samples were vortexed again, then centrifuged at 4500 x g for 10 min at 4 °C. The top organic layer and the lower aqueous layer of the supernatant were separated and the organic layer was evaporated under nitrogen and reconstituted in 95 µL chilled isopropanol.

The resulting organic phase (lipids) of each sample was analysed in triplicate with lipids chromatographically separated using a 12-minute linear gradient. Chromatography was performed using an ACQUITY™ Premier UPLC™ (Waters Corp., Milford, MA, USA) configured with a flow through needle. Separations were performed using an ACQUITY Premier CSH, 1.7 μm, 2.1 mm x 100 mm C_18_ column (Waters Corp., Milford, MA, USA) with a linear gradient applied from 50 to 99% (v/v) solvent B in 11 min using a flow rate of 400 µL/min. Re-equilibration of the system was then achieved in 1 min, providing initial starting conditions of 50% solvent B. Solvent B consisted of 900/90/10 IPA/ACN/1 M aqueous ammonium formate in 0.1% (v/v) formic acid while solvent A consisted of 600:390:10 (v: v:v) acetonitrile: water:1 M aqueous ammonium formate in 0.1% (v/v) formic acid. The eluent from the column was directly coupled to a SYNAPT XS QTOF mass spectrometer (Waters Corp., Wilmslow, UK). Data were collected in positive and negative electrospray mode, with the capillary voltage set to 3.0 kV and 2.5 kV respectively. The cone voltage was set to 30 V, source temperature of 120 °C with the cone gas (nitrogen) flowing at 50 L/h. Desolvation gas temperature and nebulisation gas (nitrogen) flow were set to 600 °C and 800 L/h respectively. The ion mobility settings comprised of the ion mobility T-wave velocity at 650 m/s, pulse height of 40 V and drift gas (nitrogen) flow rate of 180 mL/min. and data acquired over a mass range of 50-1200 Da. The time of flight (TOF) was calibrated over the mass range 50-1200 Da using sodium formate, whilst the ion mobility device was calibrated (collision cross-section [CCS] range of 130–306 Å^2^) the Major Mix IMS calibration kit (Waters Corp., Wilmslow, UK), which allowed for CCS values to be determined. All the data was collected in ion mobility mode and utilised the acquisition mode, HDMSE, providing both precursor and corresponding fragment ion information. Data were collected as continuum data, with a low collision energy (4 eV) for precursor whilst a collision energy ramp was employed (19–45 eV) to provide fragment ion data. For lipidomics, acquired mass spectrometry data were processed in Progenesis QI, using default parameters and M + H, M+NH4, M + Na, and M + K adducts in positive ionisation mode and M-H, M + Cl, and M + FA-H adducts in negative ionisation mode, and searched against the LipidMAPS, HMBD, and Progenesis MetaScope libraries using the MetaScope search plug-in.

### Data analysis

Clinical data was compared between groups and visualised using R 4.2.1 [11]. Comparisons were conducted between both waves, the very mild group and all other groups for MCSS, and the moderate group and all other groups for WHO severity score. The moderate group was chosen as a comparator for the WHO severity score due to the lack of patients in the mild group. Comparisons were conducted using Wilcoxon rank-sum tests with Benjamini-Hochberg corrections for multiple comparisons as data were not normally distributed when assessed by Shapiro-Wilk tests.

Relative normalised abundance data was scaled for further analysis by log_10_ transformation (for proteomics data) and Pareto scaling. Normality was assessed by density plots after scaling. Significantly altered proteins (fold change > 2, *p* < 0.05 by unpaired two-sided Welch’s t-test with Benjamini-Hochberg corrections for multiple comparisons) between the different patient phenotypes were selected and a heatmap of these proteins was generated using R 4.3.1, *protti* and *ComplexHeatmap* [11]. Lipidomics data was analysed for significant changes across patient groups using principal components analyses and volcano plots, performed using MetaboAnalyst 6.0 after Pareto scaling. Significantly altered compounds identified by heatmap and principal components analysis (PCA) loadings from lipidomics data were putatively identified using LipidMAPS, HMBD, and Progenesis MetaScope, including the MetaScope CCS library. For proteomics data analysis targets of the depletion step were excluded.

## Results

### Cohort demographic characteristics

The ManARTS COVID-19 cohort is an opportunistically enrolled patient population of 222 participants with clinical data regarding severity and admission date. The majority (68%) are male with a median age of 63 (Table 1). It is ethnically diverse, with a higher representation of Black, Asian and minority ethnic groups compared to the general UK population (Figure S2). Only 5% were current smokers with 40% never having smoked, consistent with patterns of smoking behaviour in older adults in the UK in the 2022 census [29]. Participants also tended to have a higher body mass index (BMI) compared to the general population, reflecting the older age of the cohort [30]. Most participants were admitted in the first wave of the pandemic in the UK between March and early September 2020 and are representative of critical care patients with COVID-19 at that time (Figure S1).

**Table 1:** ManARTS COVID-19 Cohort demographics. Obesity was defined according to the WHO classifiers [31]

Participant Characteristics	*N* (%)	Participant Characteristics	*N* (%)
**Sex**		**Wave**	
Female	69 (31%)	Wave 1 (10/3/20–2/9/20)	152 (68%)
Male	151 (68%)	Wave 2 (3/9/20–10/1/21)	70 (32%)
Missing data	2 (1%)		
**Age**		**BMI**	
Median	63	Median	29
Mean	61	Mean	30
0–49	42 (19%)	Normal Range	50 (23%)
50–59	58 (26%)	Underweight	5 (2%)
60–69	52 (23%)	Overweight	67 (30%)
70–79	42 (19%)	Obese	65 (29%)
80+	28 (13%)	Morbidly Obese	16 (7%)
		Missing data	19 (9%)
**Ethnicity**		**Smoking History**	
Black	4 (2%)	Current	11 (5%)
Mixed/Other	11 (5%)	Ex-smoker	63 (28%)
Other Asian	8 (4%)	Never	87 (39%)
South Asian	18 (8%)	Missing data	61 (28%)
White	166 (75%)		
Missing data	15 (7%)		

**Fig. 1:**
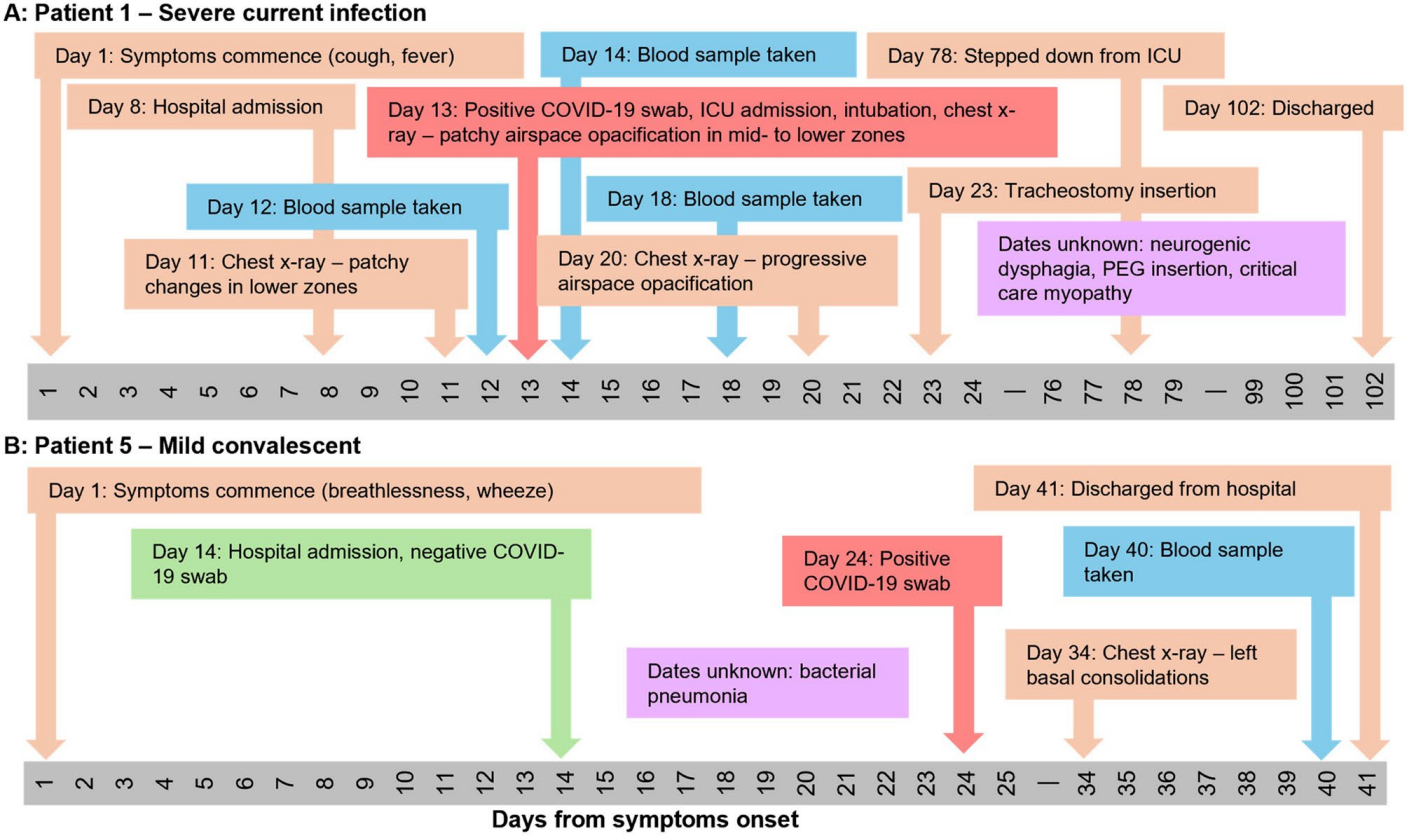
Example ManARTS COVID-19 cohort patient journeys. **A**: Patient 1 with a severe COVID-19, where blood samples were taken at multiple time points within a six-day timespan when the patient had a positive swab test for SARS-CoV-2. **B**: Patient 5 with a mild nosocomial SARS-CoV-2 infection where blood samples were taken prior to discharge. ICU – intensive care unit; PEG – percutaneous endoscopic gastrostomy

Initially an in-depth analysis of the patients’ journeys during their hospital stay was undertaken to understand heterogeneity in the cohort with regards the time of symptom onset, diagnosis of COVID-19, length of the hospital stay, coinfections, comorbidities, types of treatment, and eventual outcome (Fig. 1, Figure S13). Since serum and plasma samples were opportunistically collected, time of sampling was not linked to other types of biomarker analysis undertaken as part of routine monitoring of patients. Patient 1 is an example of a patient with a severe infection (Fig. 1A) who had a history of diabetes and was admitted to the hospital eight days after symptom onset. On day 11 after symptom onset there was evidence of patches in the lower lungs following x-ray and on day 13 following a positive SARS-CoV-2 swab test the patient was admitted to the ICU. Following a prolonged hospital stay of 102 days they were discharged. For this patient blood samples were taken at multiple time points during the active infection period, both before and after ICU admission. In contrast patient 5 had a hospital acquired SARS-CoV-2 infection, having been admitted due to bacterial pneumonia and only having a positive SARS-CoV-2 swab test 24 days after admission (Fig. 1B). Their blood sample was collected on day 40 immediately prior to discharge following a negative SARS-Cov-2 swab and thus represents a convalescent patient.

Using this analysis of patient journey’s the cohort was stratified into those patients where banked serum and plasma samples were concurrent with an active infection (*n* = 122), or when patients were convalescent (*n* = 74) (Figure S5B). An active infection was defined as being within 14 days of a patient having either a positive SARS-CoV-2 swab test, or chest X-ray presentation consistent with SARS-CoV-2 infection [29]. Convalescence was defined as where samples were taken more than 21 days after diagnosis and after discharge. In some instances, blood samples were taken from hospitalised patients more than 14 days after the diagnosis of a SARS-CoV-2 infection. The clinical histories of these patients were reviewed to ascertain their infection status (i.e. either having a current infection or who were convalescent).

### Clinical characteristics of COVID-19 infection and biomarker analysis

**Fig. 2:**
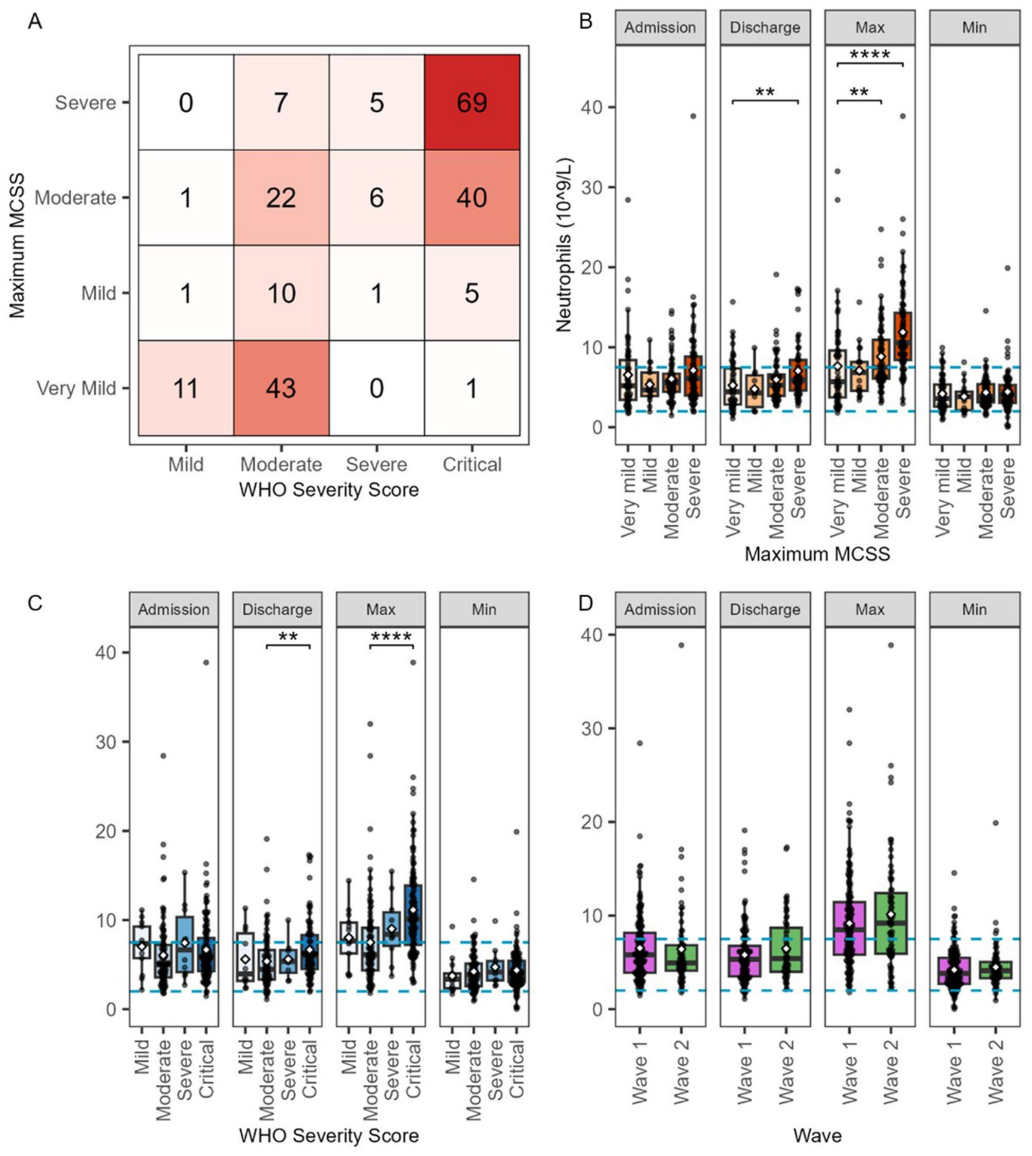
Clinical characteristics of the ManARTS COVID-19 cohort. **A**: comparison of maximum MCSS and WHO severity score. B-D: Boxplots of first, last, lowest and maximum recorded neutrophil counts, stratified by maximum MCSS (**B**), WHO severity score (**C**) and wave (**D**). ◇ = mean; --- = normal range; *=*p* < 0.05, **=*p* < 0.01, ***=*p* < 0.001 (Wilcoxon rank-sum tests with adjusted p value after Benjamini–Hochberg correction). Normal ranges were those used by the Manchester University NHS Foundation Trust at the time of sample analysis

The severity of SARS-CoV-2 infection was assessed in two different ways (Fig. 2A). The first was using the maximum Manchester COVID-19 Severity Score (MCSS) (Figure S4), an approach developed to support clinical decision making during the patients’ hospital stay. The second was a retrospective application of the WHO severity score (Figure S3). In general, most patients analysed were classified as either moderate or critically ill by the WHO score, whilst the MCSS score classified a significant proportion of the WHO moderate group as very mild (Fig. 2A).

The severity scores were then used to stratify 137 patients (119 with active infections and 18 convalescent patients, Figure S5B) for whom data for a variety of clinical cellular and biochemical markers were available (Figure S6-Figure S12), White blood cell counts were significantly altered between patients with different disease severity with an overall increase in white blood cell count during active infection (Figure S10 E1-E3), driven by increases in neutrophils (Fig. 2B-D) and eosinophils (Figure S10 A1-A3). The maximum neutrophil counts were significantly elevated in patients with more severe disease but in contrast, lymphocyte and monocyte counts were decreased. Indeed, the lymphocyte count was below normal for most patients, irrespective of severity. Many of the differences were still evident even on discharge, consistent with the majority of these patients still having an active infection but being well enough to return home.

Of the biochemical markers circulating levels of the acute phase protein, C-reactive protein (CRP) showed a marked increased in patients admitted with more severe disease, and although this was markedly lower at discharge it remained above the normal healthy level of 5 mg/L (Figure S6). This reflects the fact that patients with an active infection had a mean CRP of 74.5 mg/L (median = 46.5 mg/L) but was much lower in convalescent patients being (mean = 35.1, median = 21.0) (Figure S14). Markers of liver function were also significantly altered; albumin levels fell below normal in most patients and was more marked in patients with more severe COVID-19 (Figure S11, A1-A3). Other liver markers such as bilirubin, globulins, and alanine transaminase, increased in patients with more severe disease as did platelets and D-Dimer (Figure S12), However, the activated partial thrombin time (APTT) decreased (Figure S12) and haemoglobin levels were below normal in most patients at all timepoints, including at discharge (Figure S8). Some markers, such as creatinine, APTT, alkaline phosphatase, and eosinophils, also showed significant alterations between patients admitted to hospital during wave 1 compared to wave 2 of the pandemic.

### Pilot biomarker analysis using multiomics methods

A pilot analysis was undertaken using lipidomic and proteomic profiling using a novel, high throughput LC-MS-MS workflow. It was applied to selected patients to assess is utility and reproducibility and explore whether comparing patients experiencing a current SARS CoV-2 infection (with either mild or severe disease) with convalescent patients (who had experienced a mild SARS CoV-2 infection) was an effective approach to patient stratification (Table 2).

**Table 2:** Patient samples used in pilot lipidomics and proteomics analyses

Patient No.	Day SARS-CoV-2 infection established	Day of samples [sample no]	Max MCSS	WHO Severity Score	Status at time of sampling	Lipidomics	Proteomics
**Group 1: Severe current infection**							
PT1	23/11/2020	03/11/2020 [1.1]	Severe	Critical	Current infection	✓	✓
		05/11/2020 [1.2]			Current infection	✓	
		09/11/2020 [1.3]			Current infection	✓	
PT2	11/04/2020	29/04/2020 [2.1]	Severe	Critical	Current infection	✓	✓
		01/05/2020 [2.2]			Current infection	✓	
		01/05/2020 [2.3]			Current infection	✓	
**Group 2: Mild current infection**							
PT3	13/09/2020	17/09/2020 [3]	Very mild	Not assigned	Current infection	✓	
PT4	17/09/2020	22/09/2020 [4]	Mild	Not assigned	Current infection	✓	
**Group 2: Mild convalescent**							
PT5	16/05/2020	01/06/2020 [5]	Mild	Not assigned	Convalescent	✓	✓
PT6	09/04/2020	27/08/2020 [6]	Mild	Not assigned	Convalescent	✓	✓

**Fig. 3:**
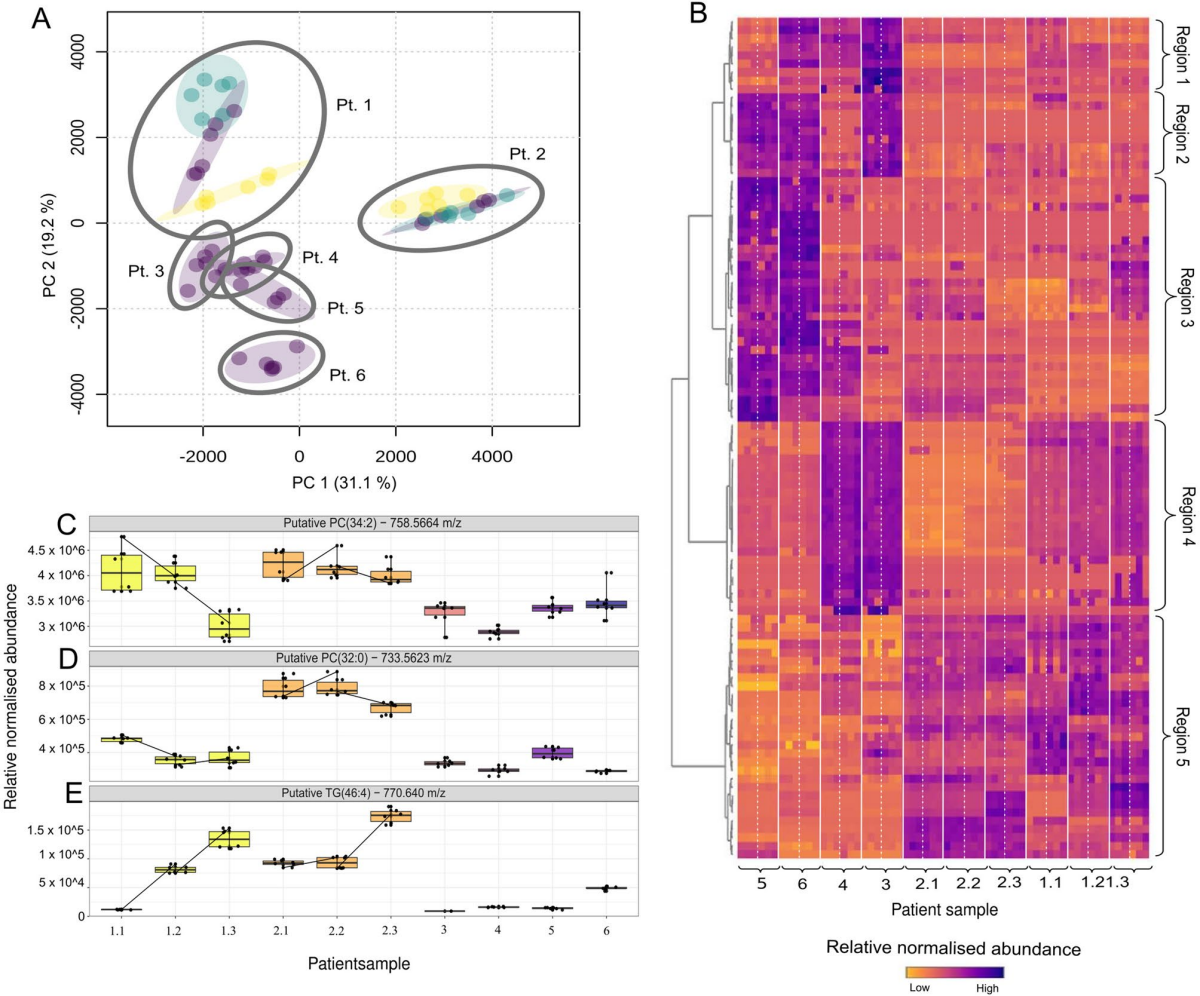
Serum lipidome profiling. **A**: PCA scores plot of mass spectrometry features in positive ionisation mode. Blue circles: Pt 5 and 6; pink squares; Pt 3 and 4; yellow triangles: Pt 1 and 2. **B**: Heatmap of the top 100 features by ANOVA. Patient numbers with timepoints (cf. Table 2) are labelled on the bottom and patient phenotypes are labelled on the top. Patient samples are separated by solid white lines and biological replicates by dotted white lines. Heatmap regions are annotated on the right. Darker colours represent a higher relative normalised abundance. **C**, **D** and **E**: The distribution of three mass spectrometric features across samples, labelled with the retention time, followed by the mass-to-charge ratio (m/z) or neutral mass (n), and then the putative identification

For lipidomic analysis a total of 1,317 mass spectral features (each representing different molecular entities) were reproducibly identified across technical replicates in positive ionisation mode. The reproducibility and consistency of identification of mass spectral features which reflects the robustness of the sample preparation and data acquisition mode, was evaluated using PCA (Fig. 3A). This was demonstrated by the technical and biological replicates from individual patients clustering together whilst there was, as anticipated, a spread of variation between the patients.

One of the challenges of untargeted lipidomics experiments is compound annotation as features may be reproducibly detected in mass spectra and show statistically significant differences in intensity between phenotypic groups, but it may not be possible to confidently annotate them without use of an orthogonal method [32, 33]. In this case, fractionation by solubility during the extraction, retention time during the chromatography, CCS, precursor and fragment ion data could be used to contribute to the level of annotation. Even so, around ~ 30% of the features identified were not matched to a compound, and the quality of the fragment ion spectra were insufficient to confidently resolve length of fatty acyls and position of double bonds for many that were matched. Therefore, features were identified by (1) headgroup (where applicable) which was confirmed by the presence of a diagnostic headgroup ion such as 184 m/z for phosphatidyl cholines (PCs); and (2) by combined number of carbons and double bonds in associated fatty acyls (e.g., PC(34:2), triglyceride (TG) (54:3)).

PCs were amongst the most common lipid classes identified and included two types of PC showing inter-patient variability (Fig. 3C, D and E). One of these was putatively identified as PC(34:2), which is increased in patients (Pt) 1 and 2. In Pt1 the levels of PC(34:2) were reduced in a sample taken 18 days after infection symptoms first appeared and were similar to patients Pt 3–6 (Fig. 3D). The second lipid was putatively identified as PC(32:0) (Fig. 3C), which was only significantly elevated in Pt2. In contrast, the triglyceride TG(46:4) (Fig. 3D) was elevated in all samples from Pt 2 and in the two samples from Pt 1.

The heterogeneous nature of the lipidomic signature of the different patients, including those with different infection phenotypes, is evident in the heatmap of the top 100 features identified by Analysis of Variance *(*ANOVA*)* as being either up- or down-regulated (Fig. 3B). Hierarchical clustering (using Euclidean distance) of features demonstrated the analysis was generally reproducible, as evidenced by the way in which replicate samples cluster together in the heatmap analysis. This analysis also revealed five regions, with region 3 comprising features that were upregulated only in Pt 5 and 6 whilst region 4 comprised features that were only upregulated in Pts 3 and 4. Region 5 comprised features generally upregulated only in Pt 1 and 2. Clusters 1 and 2 were more heterogenous, with region 1 comprising features that are upregulated in almost all patients, whilst region 2 comprised features upregulated in only in Pts 3, 5 and 6.

**Fig. 4:**
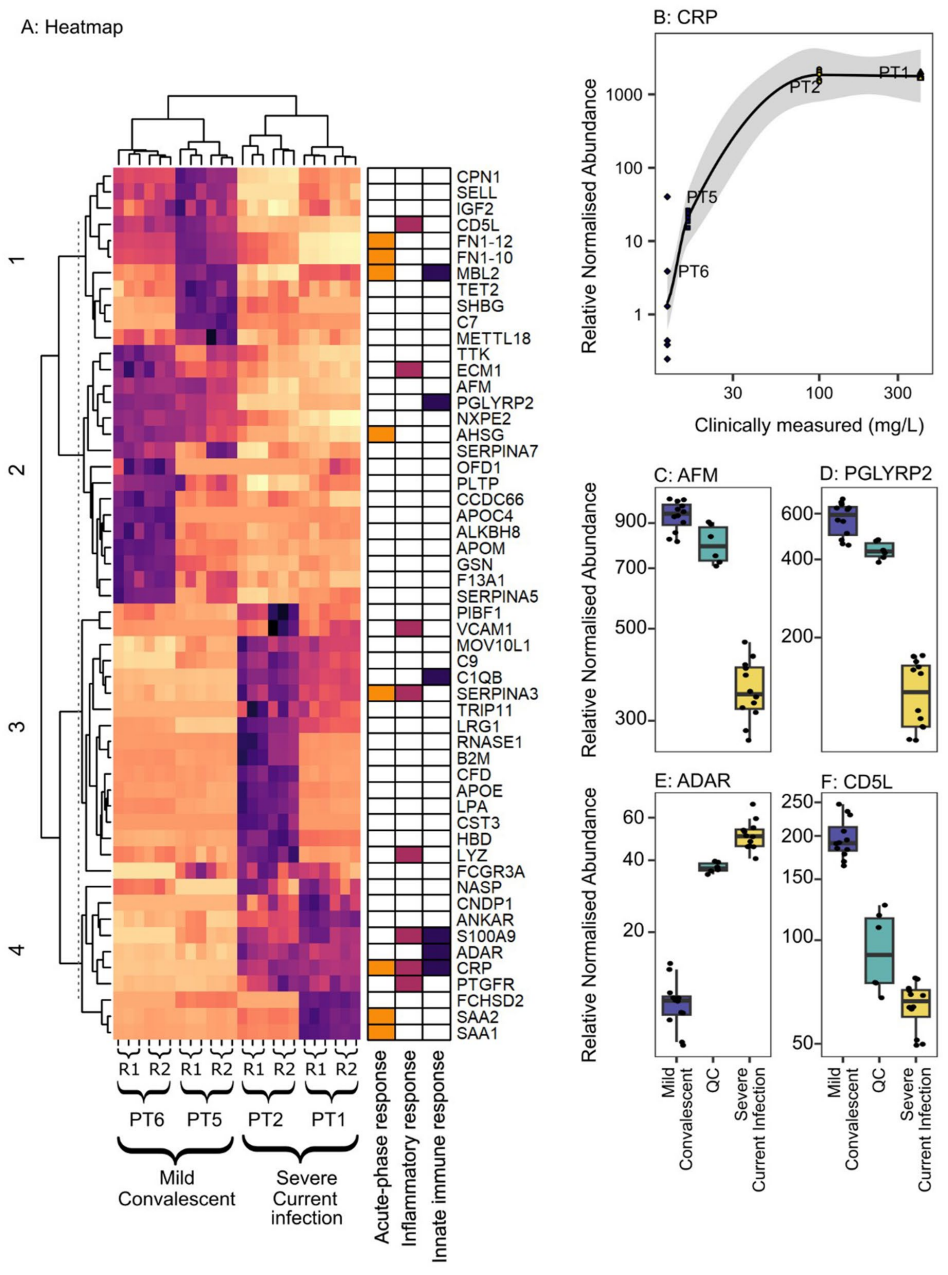
Plasma proteome profiling. **A**: Heatmap of significantly altered proteins (fold change ≥ 2, *p* < 0.05), with proteins annotated by GO terms. Darker colours represent a higher relative normalised abundance. Biological replicates of the same patient are denoted with “R1” and “R2”. Technical replicates of each biological replicate are indicated with tick marks. **B**: Relative normalised abundance from mass spectrometry correlated with clinically measured CRP at the closest available timepoint. **C**-**E**: Box-and-whisker plots of the most significantly altered proteins: **C**: CD5 antigen-like. **D**: Double-stranded RNA-specific adenosine deaminase; **E**: afamin; and **F**: N-acetylmuramoyl-L-alanine amidase. Proteins are annotated using gene names for conciseness

Proteomic analysis was undertaken using plasma samples from a subset of patients for which lipidomics was performed, comprising of two patients convalescing from mild disease and two patients with a severe current infection (Table 2). A total of 207 protein groups comprising 481 proteins were identified, with the most common gene ontology (GO) terms across significantly altered proteins by patient phenotype being those relating to innate immune, inflammatory response and acute-phase responses. *K*-means clustering of 54 proteins confirmed that, like the lipidomics analysis, the proteomics analysis was reproducible as evidenced by replicate samples clustering together in the heatmap analysis (Fig. 4A). As to be expected in a small-scale pilot, feasibility, study, patient-to-patient variability was significant but the heatmap analysis did reveal patterns of up- and down regulated proteins that differ between patients with a severe current infection compared to those who were convalescent from a mild infection. This included CRP, its relative normalised abundance in the proteomics data being correlated with CRP measured by immunoassay (Fig. 4B). Other proteins identified by proteomics as being upregulated included afamin (Fig. 4C), d-acetylmuramoyl-L-alanine amidase (Fig. 4D), double-stranded RNA-specific adenosine deaminase (Fig. 4E) and CD5 antigen-like protein (Fig. 4F).

## Discussion

The ManARTS COVID-19 cohort is one of the largest cohorts of opportunistically collected samples from early in the COVID-19 pandemic and is ethnically diverse. One its strengths is that it represents patients from a “real world” setting but this also brings limitations. For example, recruitment can make it difficult to gather necessary clinical data in a consistent manner for all patients, and it is not possible to completely standardise and control participant characteristics, unlike randomised controlled trials. Like other hospitalised cohorts it is a biased population relative to the general population as a whole with a risk of bias regarding those who consented to be recruited into the ManARTS Biobank study on COVID-19. It is not possible to ascertain the extent of this bias since not data are available regarding those who declined to participate which is a limitation of such a cohort. However, the cohort has a similar gender disparity to that observed in the ISARIC study of hospitalised patients with a preponderance of male participants, the ISARIC cohort comprising 63% males whilst ManARTS comprised 60% male [34]. This ManARTS bias towards individuals of non-white ethnicity is also consistent with individuals of non-white ethnicity being at greater risk of severe disease and mortality due to COVID-19 [34, 35] although this ethnicity gap closed after the emergence of the Omicron variant in early 2022 [36].

The pilot multiomic biomarker analysis showed the sample processing protocols and analytical pipeline gave excellent reproducibility across biological and technical replicates. It also demonstrated that that selecting serum or plasma samples taken when patients had either a current infection (mild or severe) or were convalescent was an effective means of addressing the variability in patient journeys. Similarly, the lipidomics and proteomics analysis identified changes in biomarkers associated with severity which were consistent with those identified by others. Many of the features that were significantly different between groups were phosphocholines, reflecting both their abundance in the serum lipidome and the significance of phosphocholine lipids in severe COVID-19 disease [37–39]. Similarly, changes in triglycerides (TGs) were observed which are consistent with changes TGs, low density lipoprotein and total cholesterol previously observed as both a pre-infection risk factor of severe disease and as a consequence of COVID-19 [40–42]. Likewise, proteomic analysis showed findings consistent with other work in the field, including CRP and other proteins involved in the acute-phase response, immune response, inflammation and lipid transport [1, 5, 43–46]. While the number of proteins identified is lower than when using a standard 90-minute HPLC method, the methods established in this pilot analysis were able to effectively identify potential biomarkers for COVID-19 severity. In addition, the semi-quantitative proteomics analysis of CRP in the critically ill patients was consistent with CRP concentrations determined by conventional antibody-based analysis, demonstrating that the sample workflows and data analysis used for proteomics provide good quality data. The fact that the clinically measured CRP is decreased to near-normal range in the convalescent patients supports stratification of these patients into the convalescent group and supports using this group as a control within this cohort.

As anticipated for a hospitalised patent cohort, disease severity indicated by the WHO score, a public health tool for all individuals with symptomatic COVID-19, were classified as having either severe or critical infections. In contrast, the MCSS, gave greater granularity regarding patient severity and split those classified as critical by the WHO score into two further groups. The approaches to stratification and severity scoring were then evaluated in an analysis of conventional cellular and biochemical markers. This showed, patients with a current infection had a higher CRP level and lower haemoglobin level than convalescent patients consistent with CRP being an accepted marker of severe infectious diseases [47, 48]. Similarly, liver markers were altered in a pattern consistent with COVID-19-induced liver damage, with marked decreases in the negative acute-phase reactant albumin, and increases in bilirubin, globulins, and alanine transaminase by severity [49, 50]. Similarly alterations in markers associated with coagulopathy (platelets, D-Dimer and APTT) and immune cell profiles were all characteristic of more severe COVID-19 infections [1, 51–55].

Differences in biomarkers were observed between the first two waves of the pandemic over which samples were collected. This is consistent with decreases in mortality observed in the same time period which have been attributed to changes in treatment [35]. For example, APTT decreased between waves, consistent with the use of anticoagulants, which were first advised by the International Society on Thrombosis and Haemostasis in late May 2020, after the majority (77%) of wave 1 participants in this cohort had been admitted [56]. The slightly higher levels of creatinine and alkaline phosphatase in wave 1 indicate greater disease severity affecting kidney and liver function, respectively. The slightly higher counts of eosinophils in wave 1 may reflect the use of dexamethasone later in the pandemic, characteristic of its effect of inducing apoptosis in this type of white blood cell [57, 58].

.

## Conclusions

This report demonstrates that the stratification of patients in to groups with either current infections (mild or severe) or those who were convalescent, was an effective approach for analysis of classical biochemical and immunological markers. Furthermore, a small pilot study showed this stratification was also suitable for multiomic biomarker discovery analysis. This foundation will support the application of these stratification and data curation approaches in future multiomic biomarker analysis of the cohort. with a view to gaining molecular insights into disease responses in a “real world” cohort.

## Supplementary Information

Below is the link to the electronic supplementary material.

Supplementary Material 1: Document S1 - Figures S1-S13

Supplementary Material 2: Table S2 - Protein and peptide identifications from Progenesis QI for Proteomics

Supplementary Material 3: Table S3 - Curated proteomic data following quality control

Supplementary Material 4: Table S4 - Curated lipdomic data, positive ionisation mode

Supplementary Material 5: Table S5 - Curated lipdomic data, negative ionisation mode

## Data Availability

Further information and requests for resources should be directed to and will be fulfilled by the lead contact, Professor Clare Mills (clare.mills@surrey.ac.uk). All original code for analysing proteomic data has been deposited at FigShare and is publicly available as of the date of publication. The mass spectrometry proteomics data have been deposited to the ProteomeXchange Consortium via the PRIDE [59] partner repository with the dataset identifier PXD061500 and 10.6019/PXD061500.

## Abbreviations

ANOVA: Analysis of Variance
APTT: Activated partial thrombin time
CCS: Collision cross-section
CRP: C-reactive protein
GO: Gene ontology
ICU: Intensive care unit
ESI: Electrospray ionisation
FDR: False discovery rate
FWHM: Full width at half-maximum
HPLC: High performance liquid chromatography
HDMS^E^: High definition mass spectrometry
LC: liquid chromatography
LC-MS-MS: Liquid chromatography mass spectrometry
ManARTS Biobank: Manchester Allergy, Respiratory and Thoracic Surgery Biobank
MCSS: Manchester COVID-19 Severity Score
PC: Phosphatidyl choline
PCA: Principal components analysis
PEG: Percutaneous endoscopic gastrostomy
QC: Quality control
TOF: Time of flight
TG: triglyceride
UPLC: Ultra-performance liquid chromatography
WHO: World Health Organisation
