# Supplementary material for: Characterisation of a cohort of opportunistically recruited patients with COVID-19 and approaches to patient stratification
Source: BMC Infect Dis. 2026 Mar 31;26:923. doi: 10.1186/s12879-026-13070-7 (PMC13162505; doi:10.1186/s12879-026-13070-7)
Supplement: Supplementary file 5 — Supplementary Material 5: Table S5 - Curated lipdomic data, negative ionisation mode [file 12879_2026_13070_MOESM5_ESM.docx]

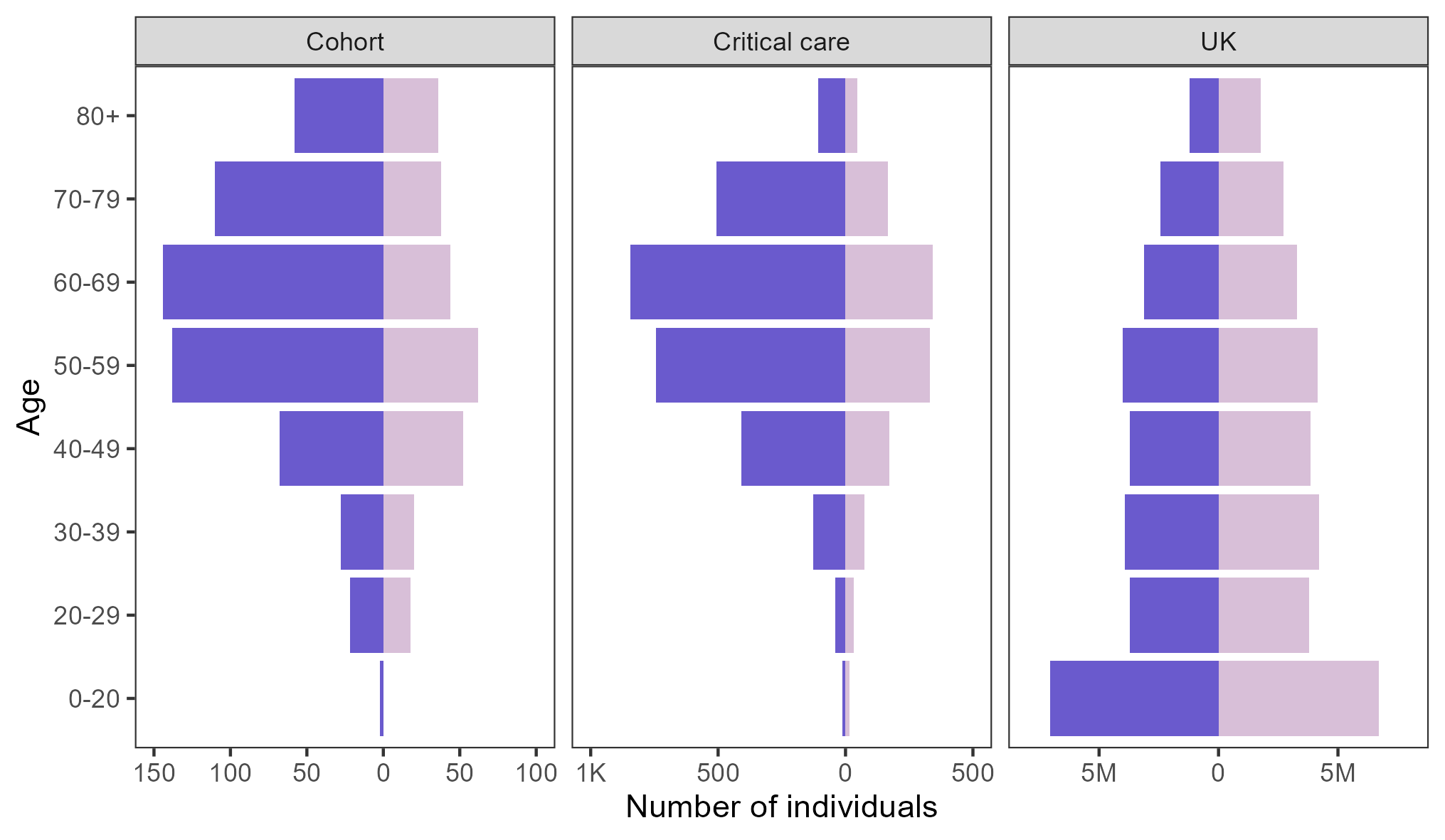


*Figure S1: Age-sex pyramids for the ManARTS COVID-19 Cohort, critical-care COVID-19 patients in the UK, and the general UK population (⬤ = Male, ⬤ = Female)^1,2^.*


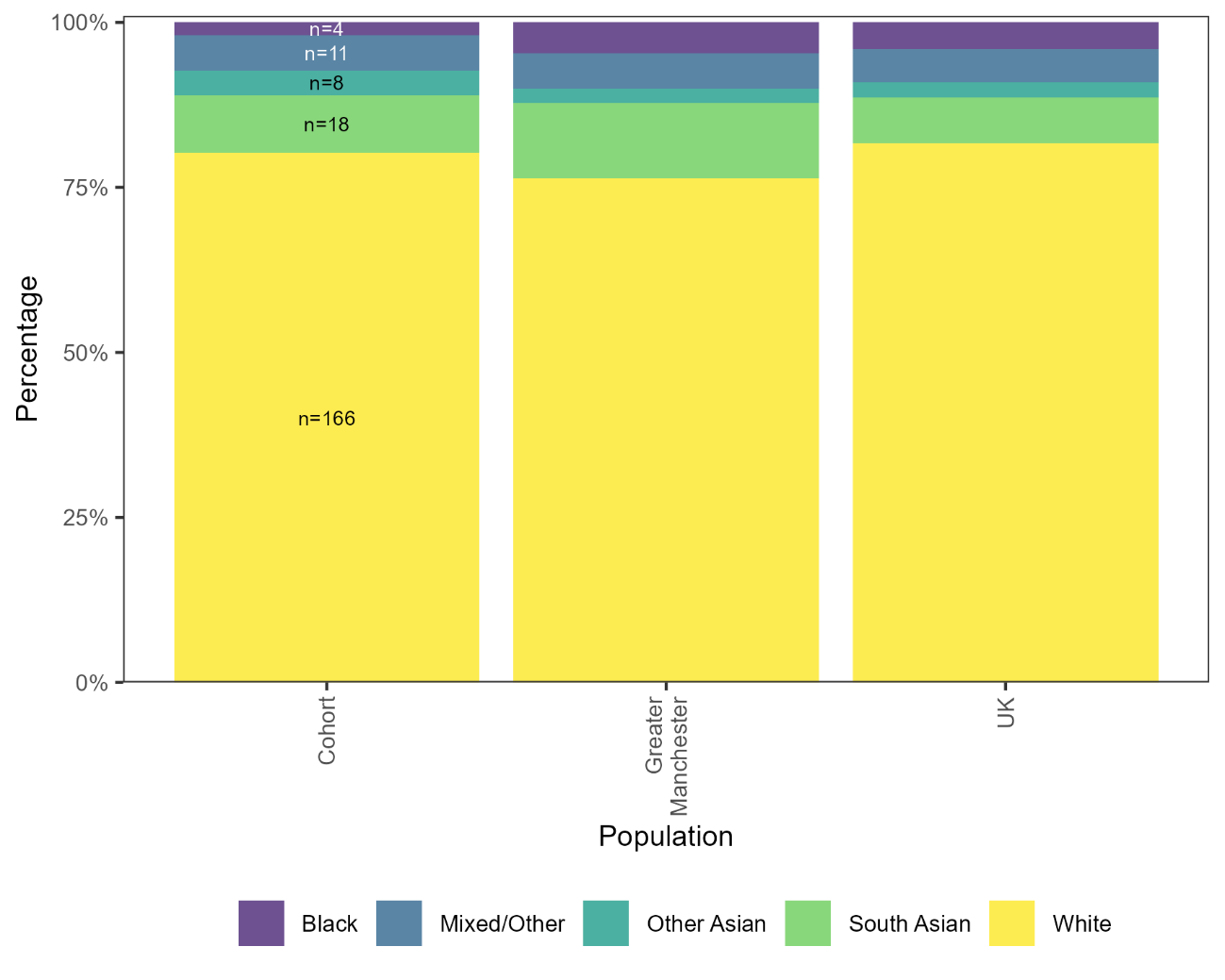


*Figure S2: Ethnicity distribution of A) the ManARTS COVID-19 cohort, B) Greater Manchester and C) the UK^3^.*


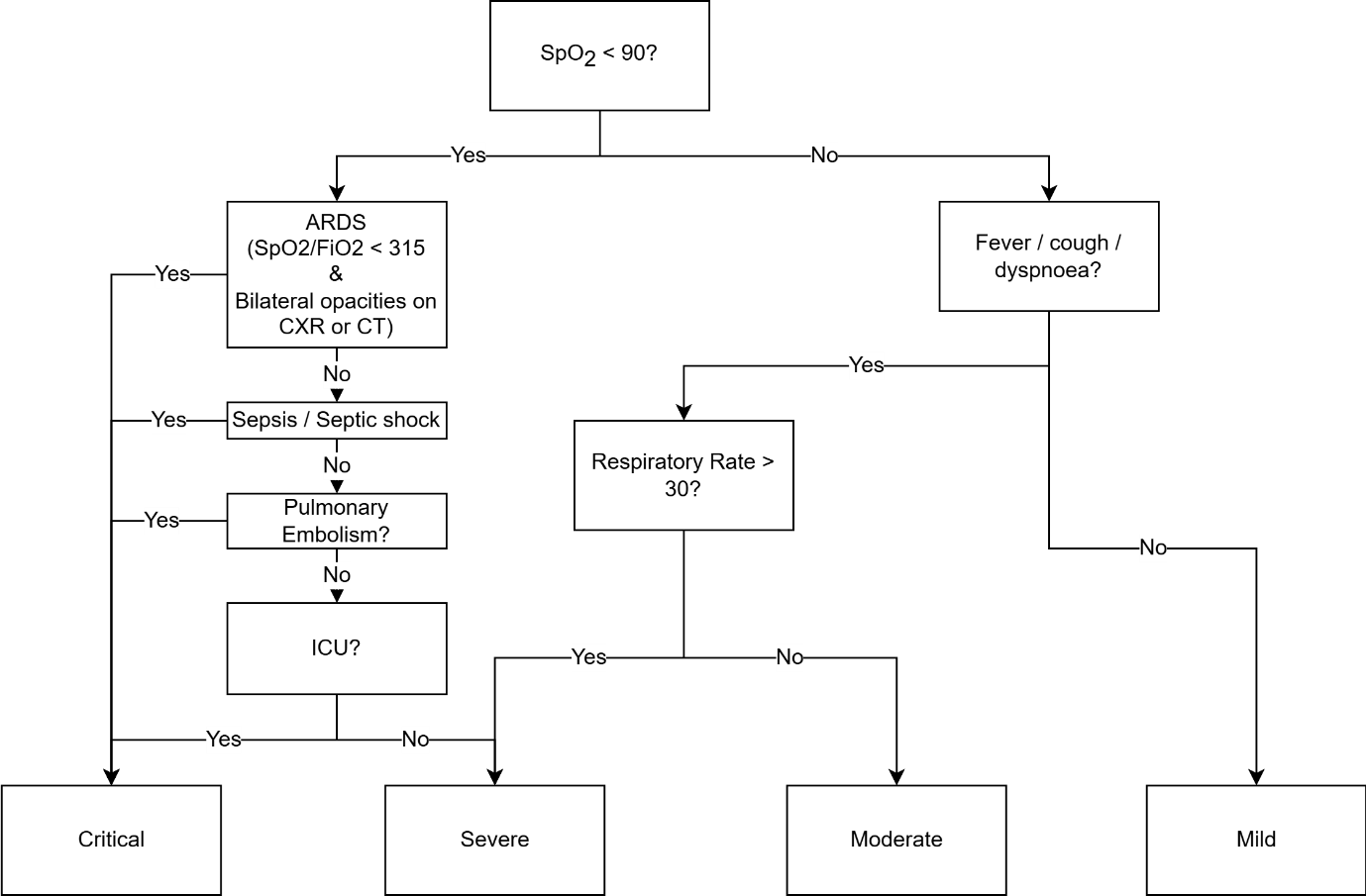


Figure S3: Decision tree for classification by WHO severity score.


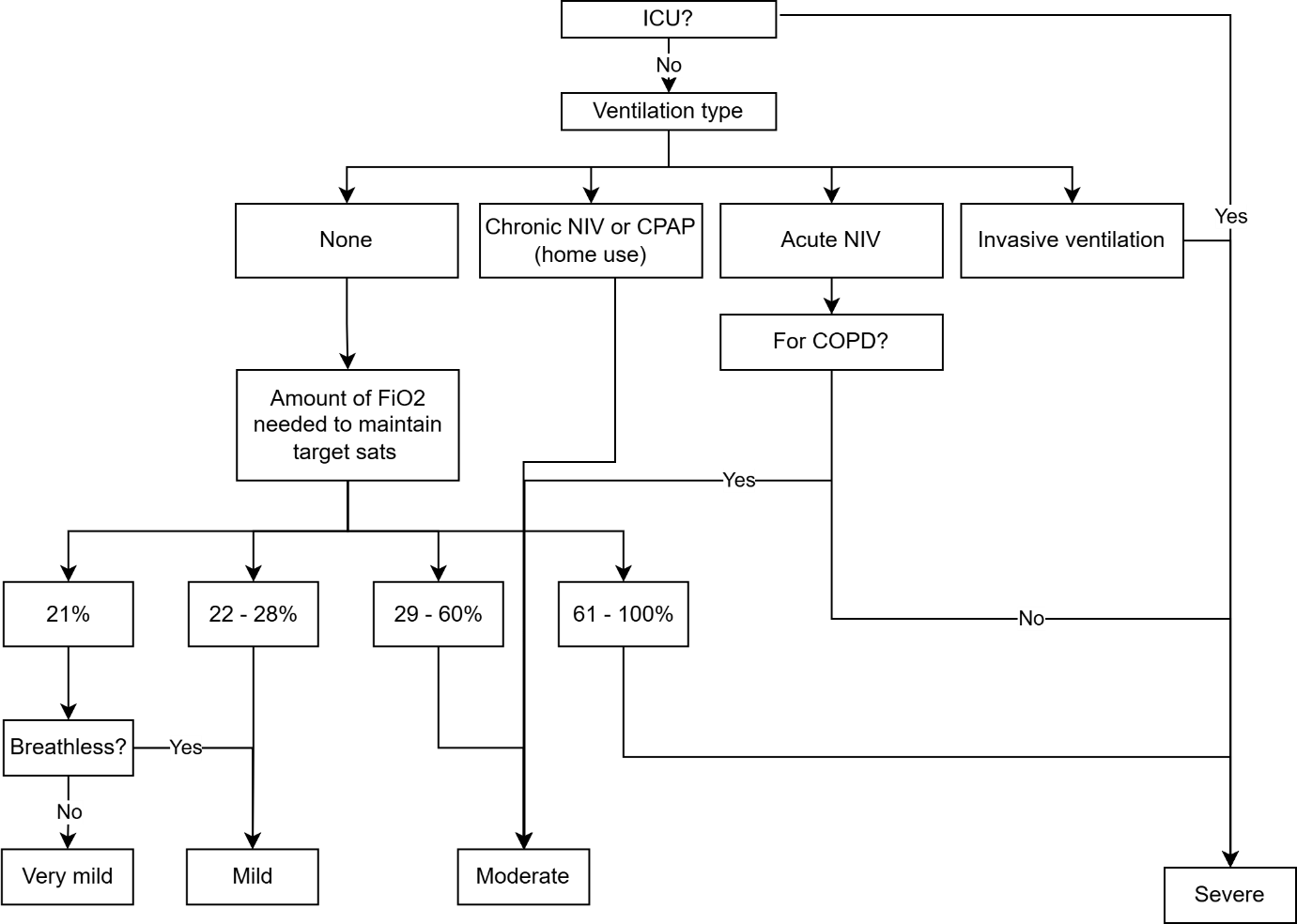


Figure S4: Decision tree for classification by MCSS.


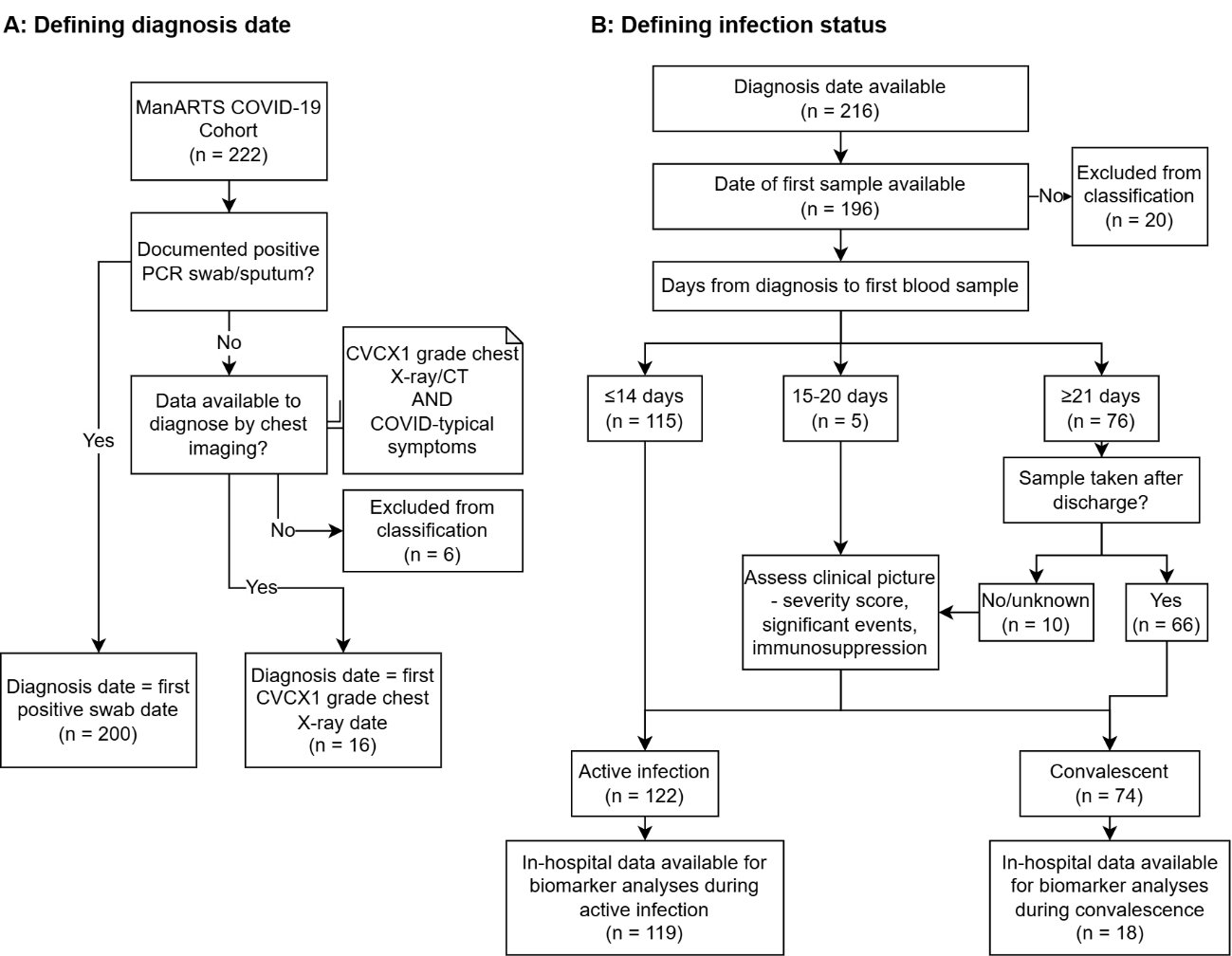


*Figure S5: Assessment of active infection at time of blood sample collection. A: Definition of diagnosis date. B: Classification of patients by infection status.*

Figure S6-Figure S12 show boxplots of first, last, lowest and maximum-recorded clinical laboratory test results, stratified by maximum MCSS (A), WHO severity score (B) and wave (C). ◇ = mean; --- = normal range; *=p<0.05, **=p<0.01, ***=p<0.001 (Wilcoxon signed-rank test). Patients with comorbidities or on medications likely to affect a particular biomarker were removed from that analysis.


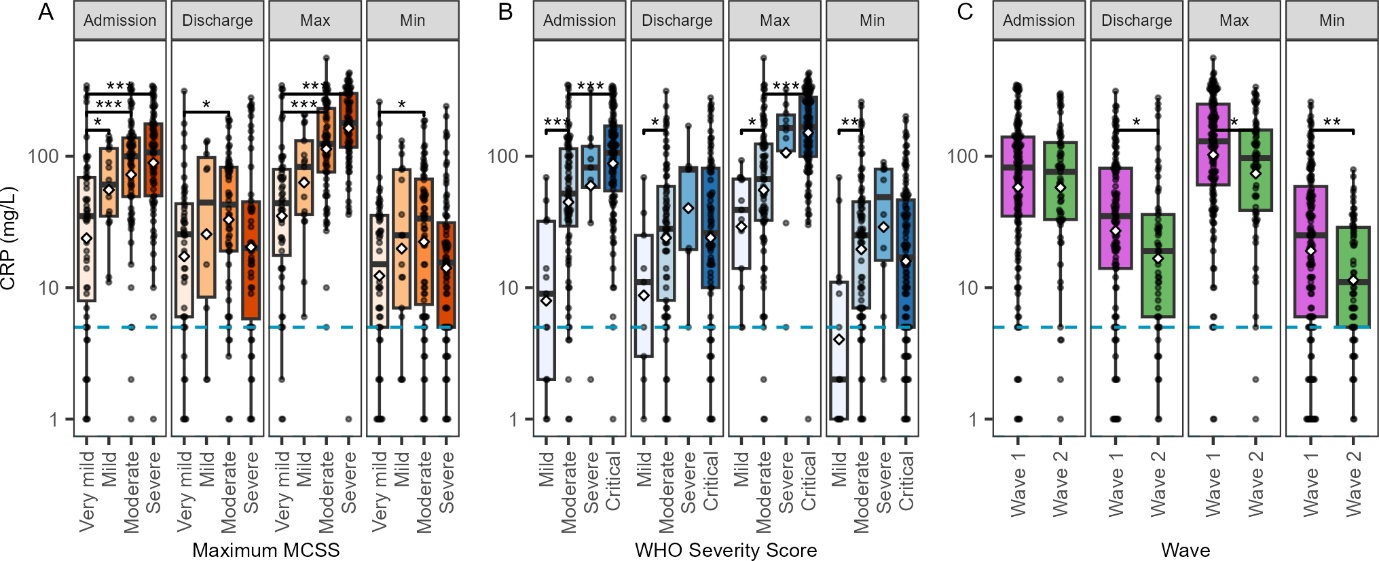


Figure S6: C-reactive protein, which is a marker of inflammation and a predictor of severe COVID-19^4^, shows significant differences by both severity and wave. Notably, it does not decrease to normal levels by discharge in the majority of patients.


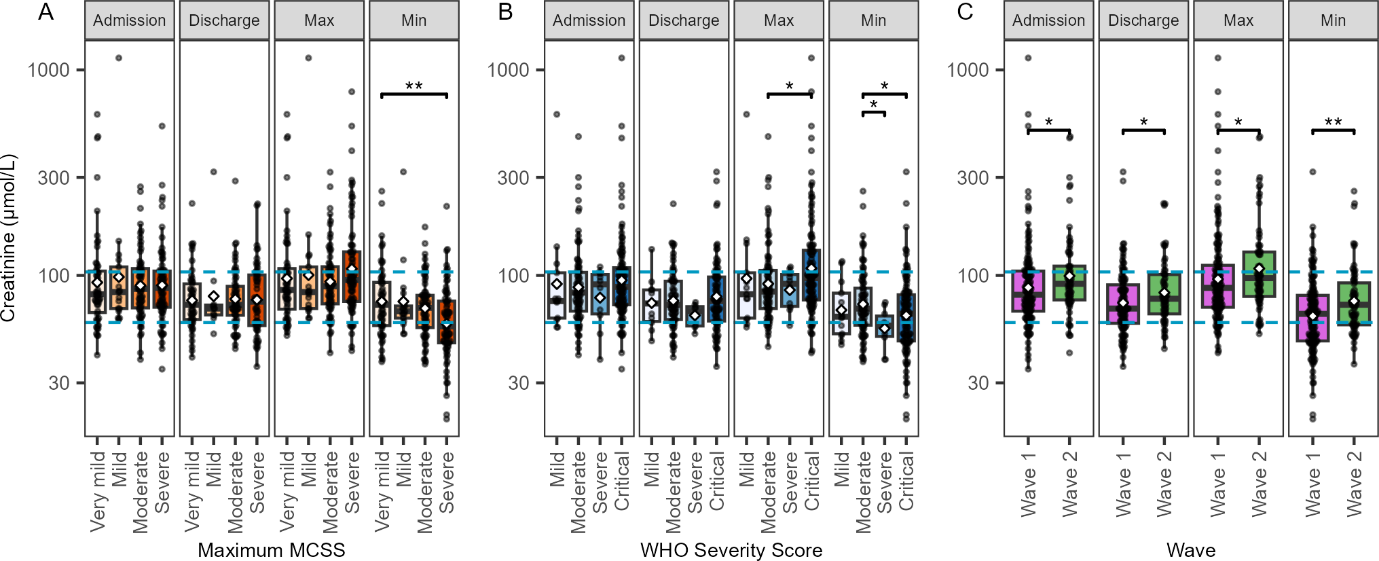


Figure S7: Creatinine, a marker of kidney damage, shows significant different by severity and across waves.


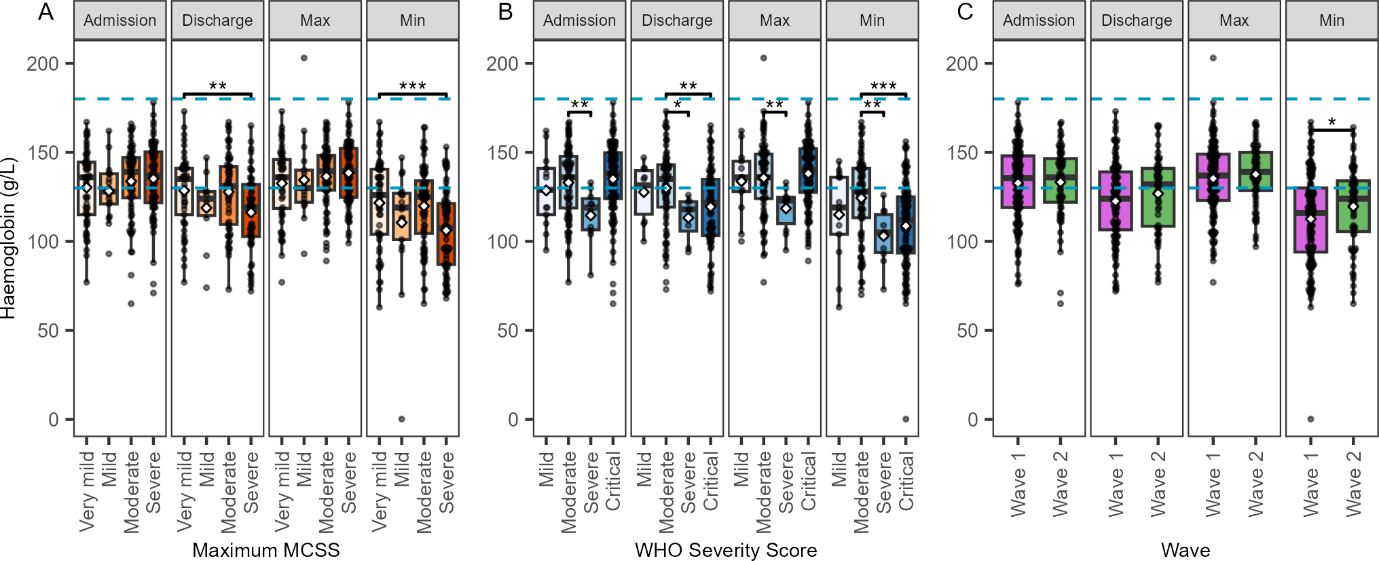


Figure S8: Haemoglobin falls below normal levels in the majority of patients regardless of severity and is markedly decreased in severe patients.


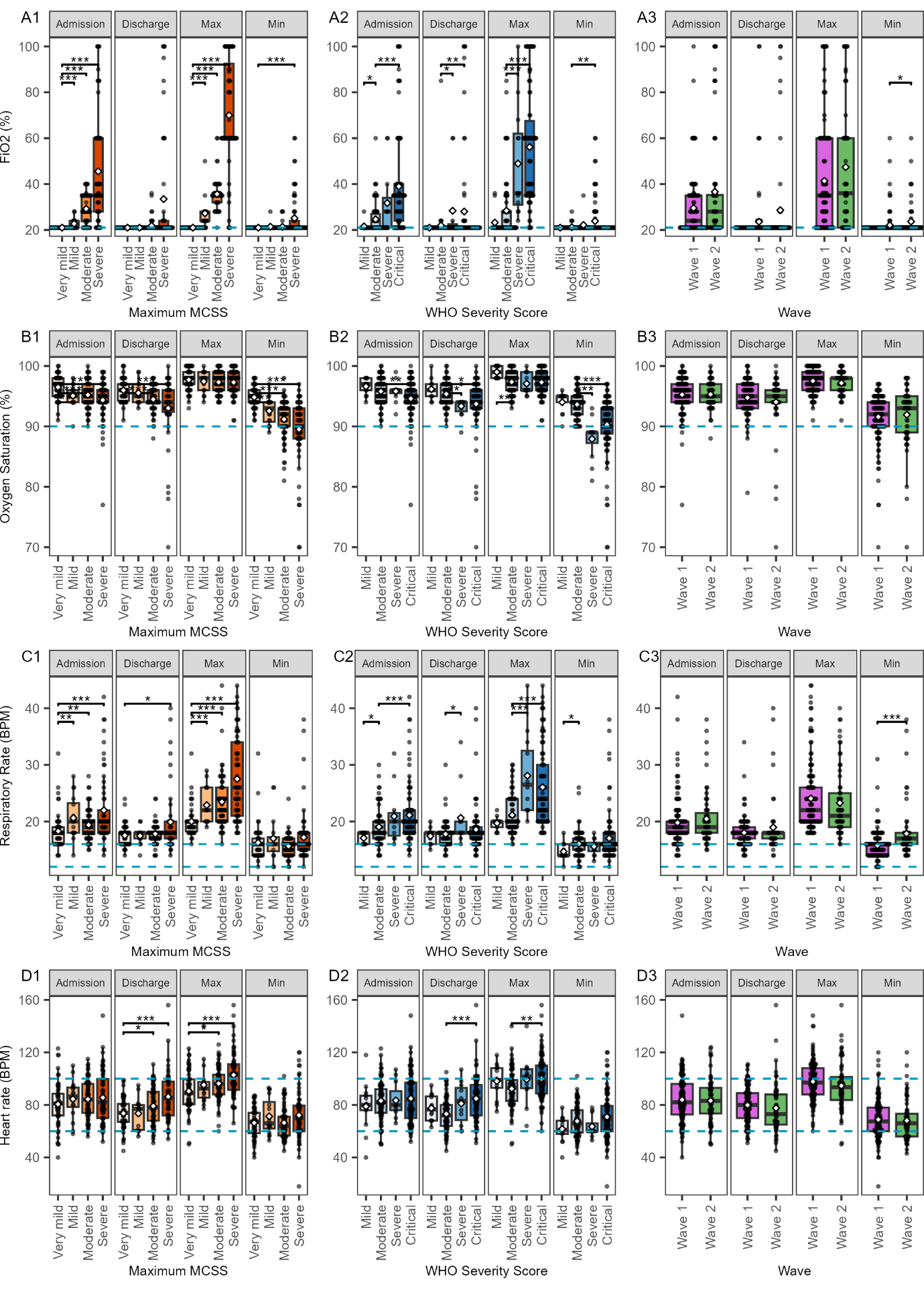
Figure S9: Respiratory markers showed significant differences across severities. FiO2 = Fraction of inspired oxygen.


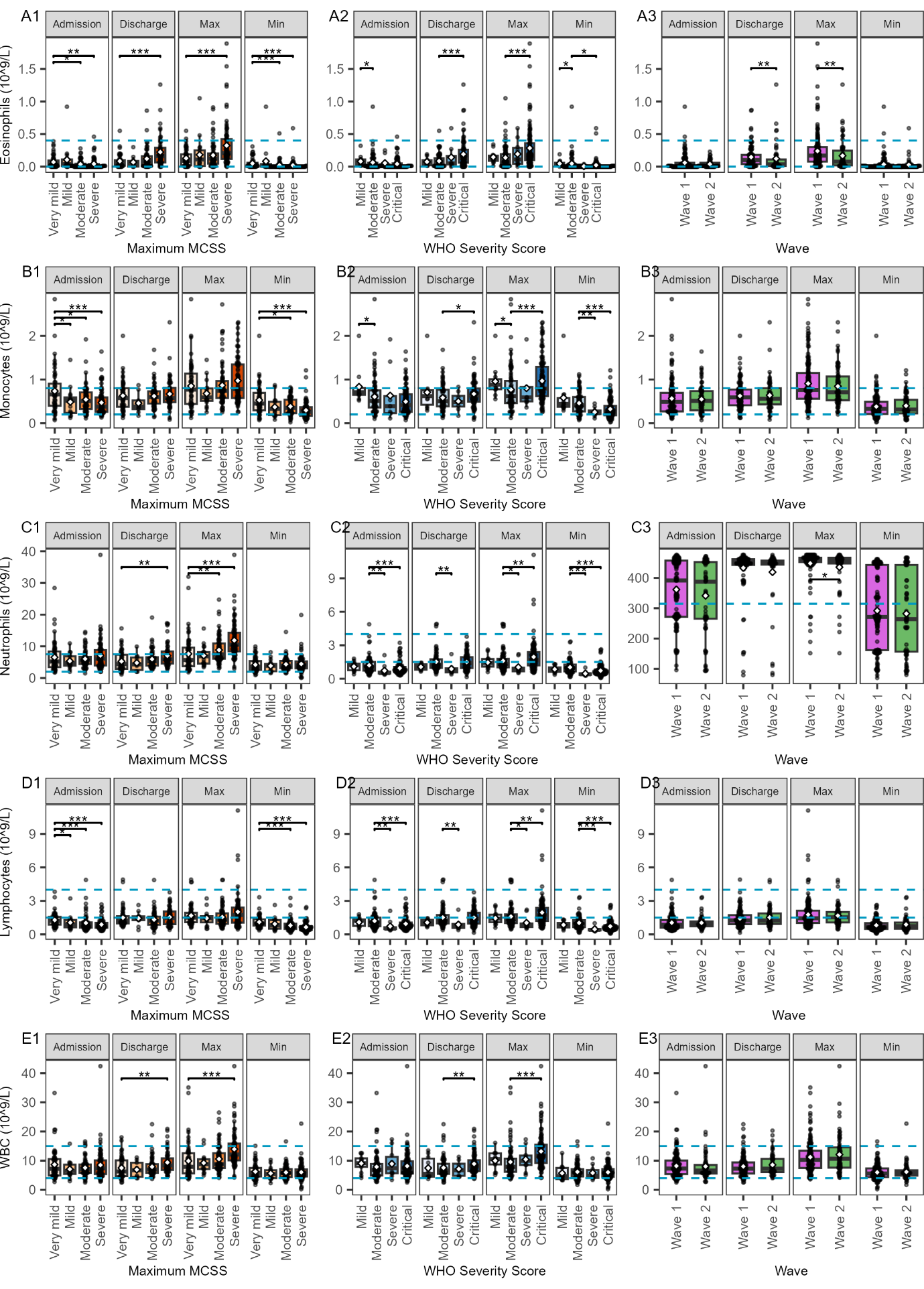


Figure S10: White blood cell counts. Neutrophil and total white blood cell counts increased with severity, while lymphocyte count decreased. Notably, lymphocyte count fell below normal level for the majority of patients regardless of severity. WBC = White blood cells.


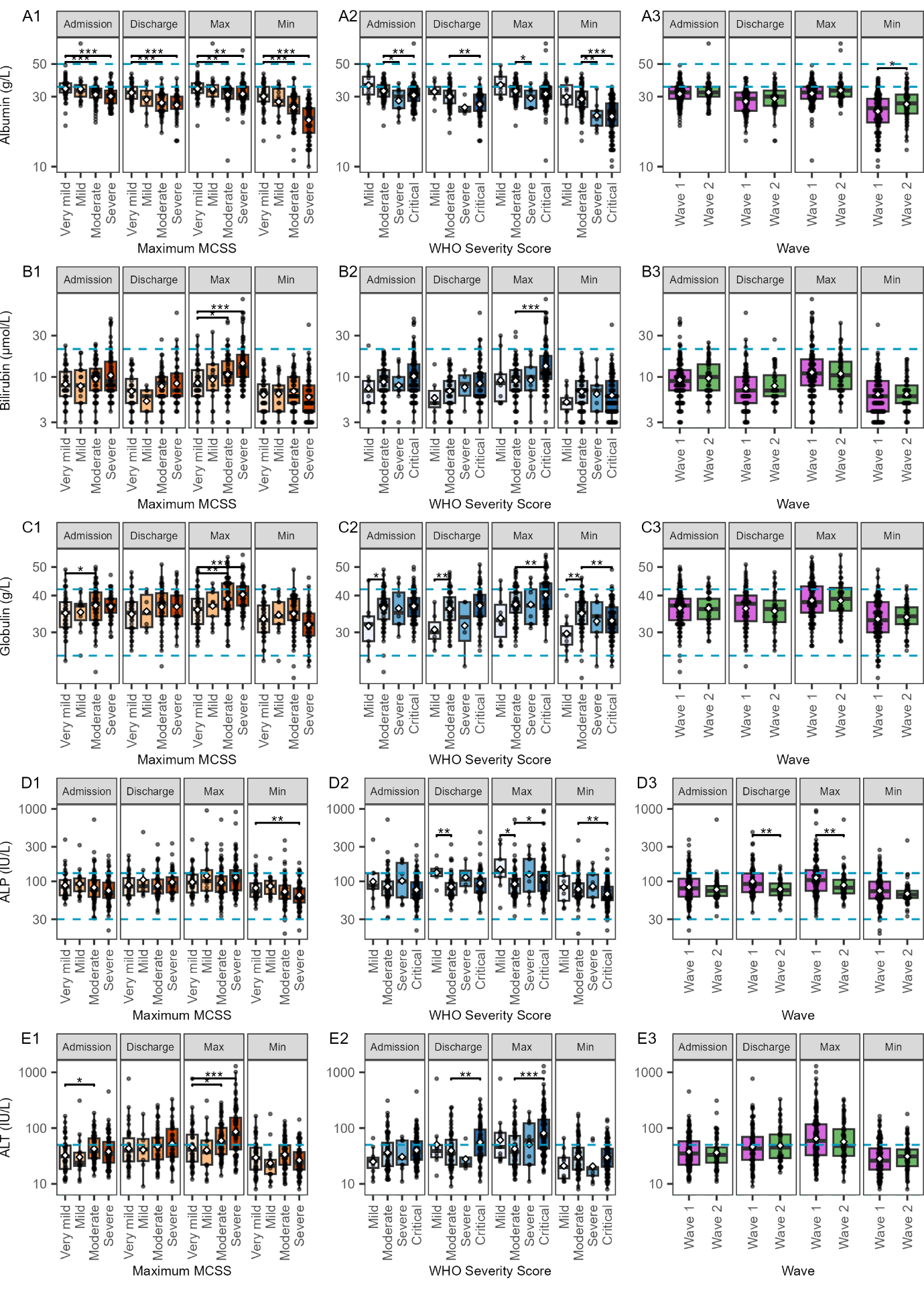
Figure S11: Liver function tests showed significant alteration across severities. ALP = Alkaline phosphatase; ALT = alanine transaminase.


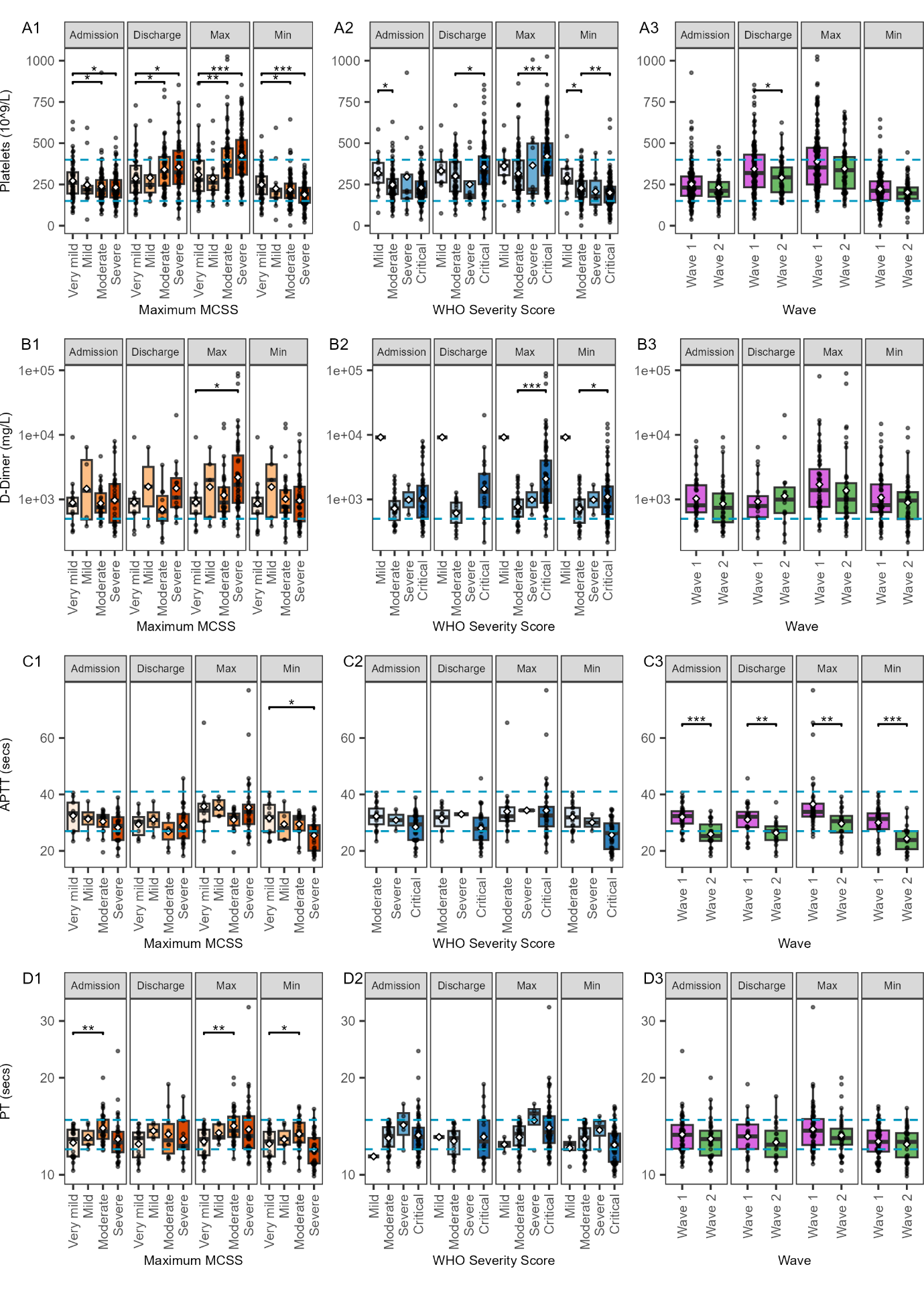


Figure S12: Clotting markers. Prothrombin time (PT), platelets and D-Dimer showed significant differences across severity, particularly when stratified by MCSS, while activated partial thrombin time (APTT) showed significant differences across waves.


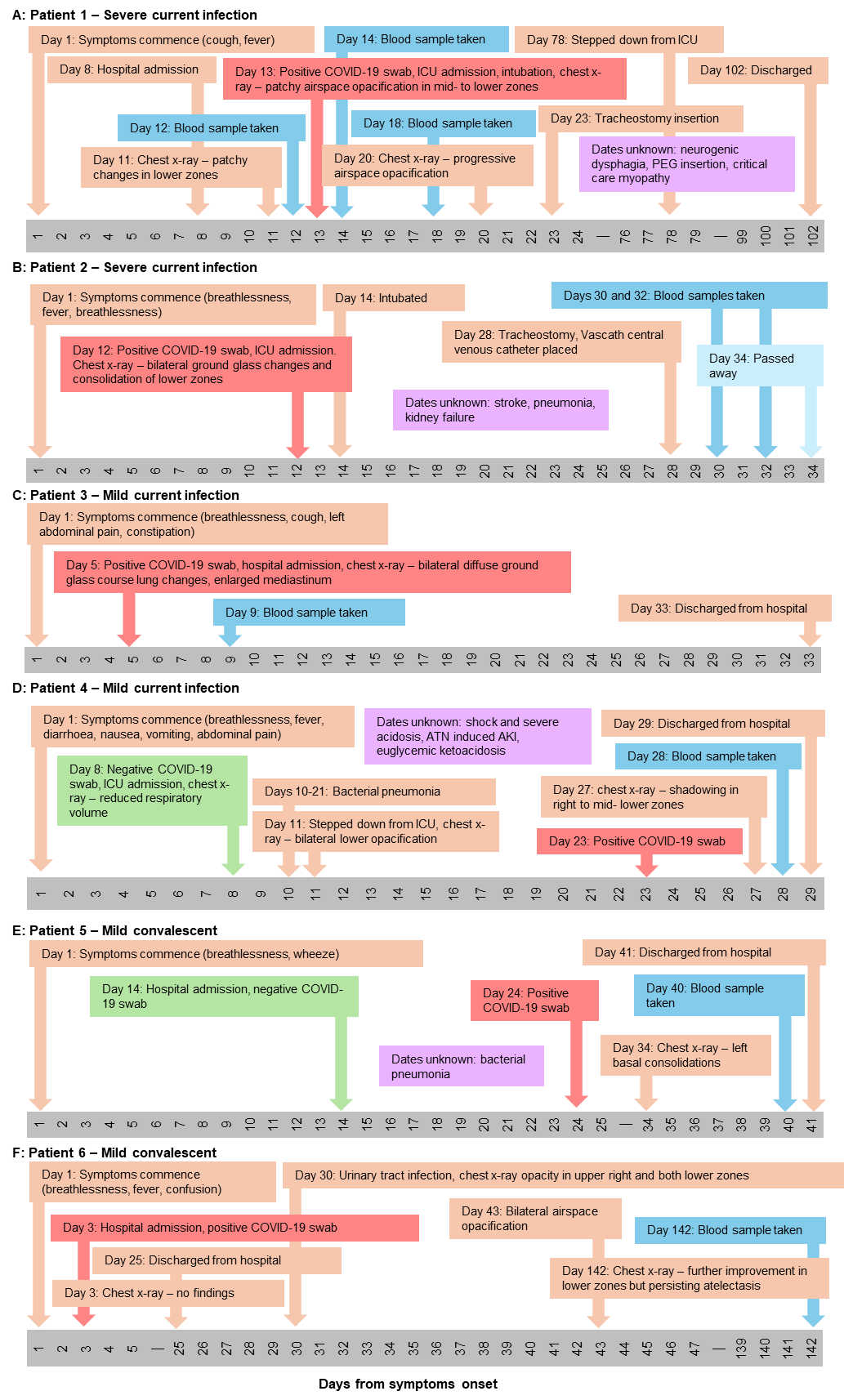


*Figure S13: Patient journeys for all patients included in the pilot mutliomics analysis. ICU – intensive care unit; PEG – percutaneous endoscopic gastrostomy; ATN: acute tubular necrosis; AKI: acute kidney injury*

Figures S14: CRP levels in patients with a current infection and those who were convalescent. Data on circulating levels of CRP were available for 115 of 199 patients with an active infection and 17 of 19 convalescent patients.


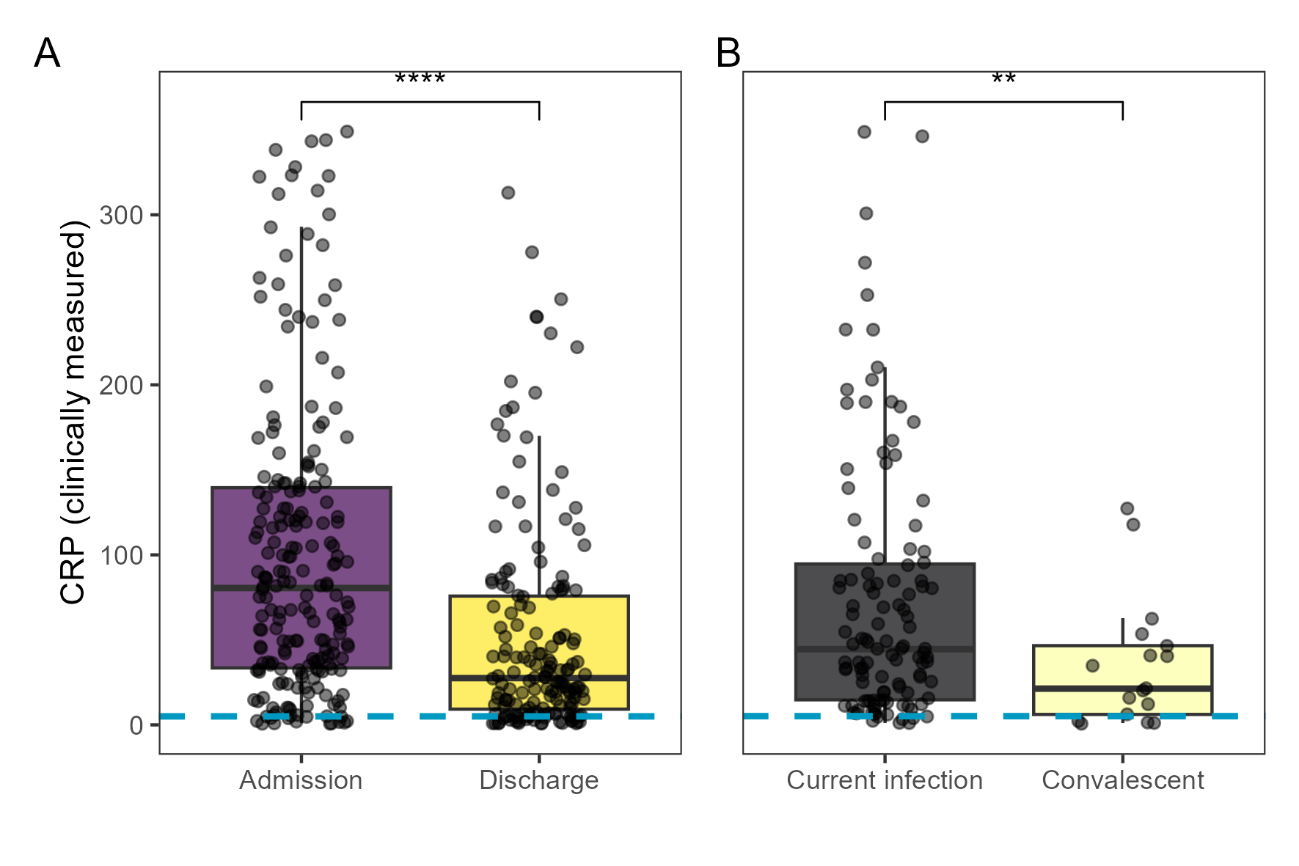

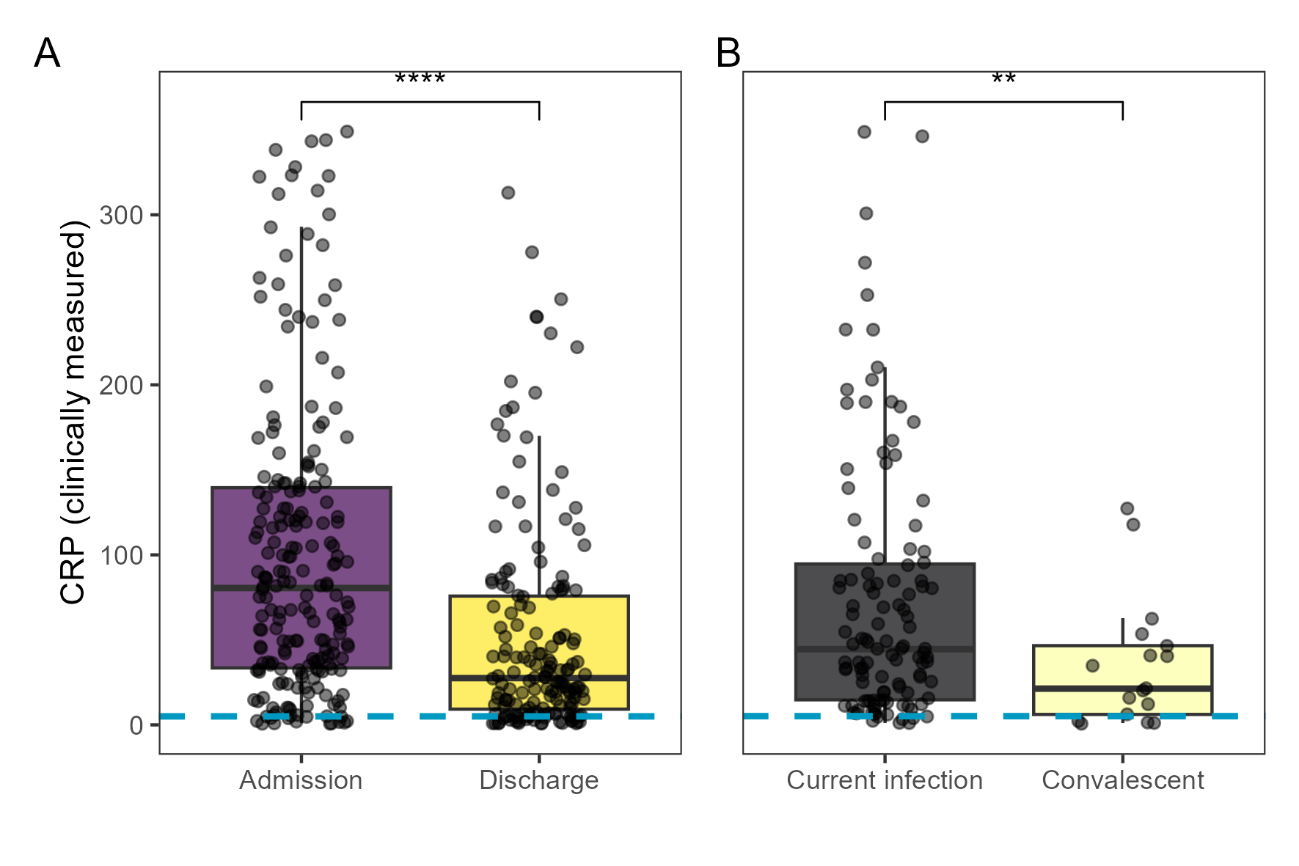


# References

1. England, P.H. (2020). Disparities in the risk and outcomes of COVID-19. 8/2020.

2. Office for National Statistics (ONS) (2 November 2022). ONS website, statistical bulletin, Population and household estimates, England and Wales - Office for National Statistics.

3. Office for National Statistics (ONS) (29 November 2022). ONS website, statistical bulletin, Ethnic group, England and Wales: Census 2021.

4. Knight, S.R., Ho, A., Pius, R., Buchan, I., Carson, G., Drake, T.M., Dunning, J., Fairfield, C.J., Gamble, C., Green, C.A., et al. (2020). Risk stratification of patients admitted to hospital with covid-19 using the ISARIC WHO Clinical Characterisation Protocol: development and validation of the 4C Mortality Score. BMJ *370*, m3339. 10.1136/bmj.m3339.
